# Altered pattern of circulating miRNAs in HIV lipodystrophy perturbs key adipose differentiation and inflammation pathways

**DOI:** 10.1172/jci.insight.150399

**Published:** 2021-09-22

**Authors:** Suman Srinivasa, Ruben Garcia-Martin, Martin Torriani, Kathleen V. Fitch, Anna R. Carlson, C. Ronald Kahn, Steven K. Grinspoon

**Affiliations:** 1 Metabolism Unit, Massachusetts General Hospital and Harvard Medical School, Boston, Massachusetts, USA.; 2 Section on Integrative Physiology and Metabolism, Joslin Diabetes Center and Harvard Medical School, Boston, Massachusetts, USA.; 3 Division of Musculoskeletal Imaging and Intervention, Massachusetts General Hospital and Harvard Medical School, Boston, Massachusetts, USA.

**Keywords:** AIDS/HIV, Endocrinology, Adipose tissue

## Abstract

We identified a microRNA (miRNA) profile characterizing HIV lipodystrophy and explored the downstream mechanistic implications with respect to adipocyte biology and the associated clinical phenotype. miRNA profiles were extracted from small extracellular vesicles (sEVs) of HIV-infected individuals with and without lipodystrophic changes and individuals without HIV, among whom we previously showed significant reductions in adipose *Dicer* expression related to HIV. miR-20a-3p was increased and miR-324-5p and miR-186 were reduced in sEVs from HIV lipodystrophic individuals. Changes in these miRNAs correlated with adipose *Dicer* expression and clinical markers of lipodystrophy, including fat redistribution, insulin resistance, and hypertriglyceridemia. Human preadipocytes transfected with mimic miR-20a-3p, anti–miR-324-5p, or anti–miR-186 induced consistent changes in latent transforming growth factor beta binding protein 2 (*Ltbp2*), *Wisp2*, and *Nebl* expression. Knockdown of *Ltbp2* downregulated markers of adipocyte differentiation (*Fabp4*, *Ppar*γ, *C/ebpa*, *Fasn*, *adiponectin*, *Glut4*, *CD36*), and *Lamin C*, and increased expression of genes involved in inflammation (*IL1****β*, *IL6*, and *Ccl20*). Our studies suggest a likely unique sEV miRNA signature related to dysregulation of *Dicer* in adipose tissue in HIV. Enhanced miR-20a-3p or depletion of miR-186 and miR-324-5p may downregulate *Ltbp2* in HIV, leading to dysregulation in adipose differentiation and inflammation, which could contribute to acquired HIV lipodystrophy and associated metabolic and inflammatory perturbations.

## Introduction

HIV lipodystrophy is the most prevalent form of acquired lipodystrophy (1). The altered fat distribution seen in HIV lipodystrophy has significant implications for metabolic risk, including cardiovascular disease (2, 3) and nonalcoholic fatty liver disease (4), which are leading contributors to morbidity and mortality in HIV. Individuals with HIV lipodystrophy can present with lipohypertrophy, with increased fat in the abdomen, viscera, and dorsocervical areas, and/or lipoatrophy, with loss of fat in the face, extremities, and subcutaneous depots. These changes are usually associated with insulin resistance, metabolic dysregulation, and inflammation. The pathogenic mechanisms of HIV lipodystrophy remain unclear. While HIV lipodystrophy was initially attributed to antiretroviral-mediated toxic effects on adipocytes, there is an increasing appreciation that the HIV and its related proteins may have direct effects on key metabolic and inflammatory pathways that may impact adipose function (5). Recent progress by our group has focused on a mechanistic hypothesis for adipose dysfunction and metabolic changes in HIV, relating to the dysregulation of *Dicer* (6), an integral protein in the microRNA (miRNA) processing pathway, and its subsequent effects on critical miRNAs regulating adipogenic pathways (7, 8).

miRNAs have important regulatory functions in adipose tissue biology. Indeed, mice lacking *Dicer* in adipocytes (Adiponectin-Cre+ *Dicer*^fl/fl^; a*Dicer-*KO) display a marked biological dysregulation of multiple miRNAs and mRNA targets associated with lipodystrophy and demonstrate a phenotype composed of multiple metabolic alterations, including insulin resistance, mitochondrial dysfunction, signs of oxidative stress, fatty liver, and premature mortality (8, 9). These phenotypic changes in the a*Dicer*-KO mice closely resemble those seen in the lipodystrophy associated with HIV (8). Interestingly, expression of *Dicer* in subcutaneous adipose tissue is markedly reduced in individuals with HIV lipodystrophy, providing a mechanistic link for overlapping metabolic features between HIV lipodystrophy and the a*Dicer*-KO mice (6, 8).

Given the downregulation of adipose *Dicer* in individuals with HIV lipodystrophy, a human physiological model that complements the animal data from the complete genetic knockout, we sought to explore whether these individuals with lipodystrophy demonstrated alterations in circulating miRNAs that might underlie the metabolic alterations in lipodystrophy (3, 10–12). Adipose tissue is a major contributor to the circulating miRNAs carried by small extracellular vesicles (sEVs), which have regulatory functions in distant tissues (13, 14). Building on prior work (13), we now comprehensively assess circulating miRNAs in individuals with HIV and lipodystrophy, individuals with HIV without lipodystrophy, and matched uninfected individuals and perform functional studies to investigate the subsequent effects of altered miRNAs on adipose tissue. We characterize a unique pattern of differentially regulated sEV miRNAs, which we show to have important gene targets in adipocytes, affecting critical adipogenic and inflammatory pathways. Thus, the current investigation identifies a potentially novel mechanism for acquired HIV lipodystrophy, highlighting key downstream consequences of reduced *Dicer* expression, systemic dysregulation of key miRNAs, and subsequent effects of altered miRNAs on genes affecting adipose regulation.

## Results

### Altered body composition and reduced adipose Dicer expression seen in HIV lipodystrophy.

To address the role of the miRNA processing pathway in the metabolic alterations caused by HIV infection, we first studied a cohort of individuals with HIV displaying lipodystrophy, individuals with HIV and no lipodystrophy, and uninfected individuals without HIV. As shown in Table 1, age and race were similar upon stratification into 3 groups (HIV/lipo, HIV/nonlipo, non-HIV). Dorsocervical adipose tissue area was largest in the HIV/lipo group and smallest in the non-HIV group (*P* < 0.004). These dorsocervical adipose tissue measurements obtained by imaging confirmed our clinical assessment for lipodystrophy in which participants with lipodystrophy were recruited for presence of increased dorsocervical fullness (Table 1). In addition, we confirmed using dual-energy X-ray absorptiometry (DXA) that limb fat (6345.4 ± 975.2 vs. 8679.7 ± 816.9 g, *P* = 0.04 by 1-way ANOVA) was lower in the HIV lipodystrophic versus HIV nonlipodystrophic phenotypes, suggesting evidence of peripheral lipoatrophy as would be expected in lipodystrophy. As previously described (6), *Dicer* expression, as determined by quantitative PCR (qPCR), was most reduced among HIV/lipo, followed by HIV/nonlipo and non-HIV (2.49[0.02, 4.88] vs. 11.20[4.83, 21.45] vs. 17.69[10.72, 47.91], median and IQR, *P* = 0.002) (Table 1). HIV-related characteristics among HIV/lipo participants and HIV/nonlipo participants differed by duration of HIV infection (24 ± 2 vs. 18 ± 3 years, *P* = 0.07), duration of ART (20 ± 2 vs. 11 ± 2 years, *P* = 0.007), duration of protease inhibitor use (16 ± 3 vs. 9 ± 2 years, *P* = 0.08), and CD8^+^ count (725 ± 115 vs. 1260 ± 164 cells/μL, *P* = 0.02) (all ranges show mean ± SEM). Overall, both HIV groups demonstrated good immunological control based upon CD4^+^ count and had similar viral loads. Respiratory quotient determined by indirect calorimetry was lowest among the non-HIV participants (Table 1). Among the HIV participants, *Dicer* expression was significantly related to CD8^+^ *P* *=* 0.51, *P* = 0.03) count and inversely to duration of ART (*P* *=* –0.60, *P* = 0.009).

### Altered pattern of circulating sEV-carried miRNAs in HIV lipodystrophy associated with reduced adipose Dicer expression.

**Figure 1:**
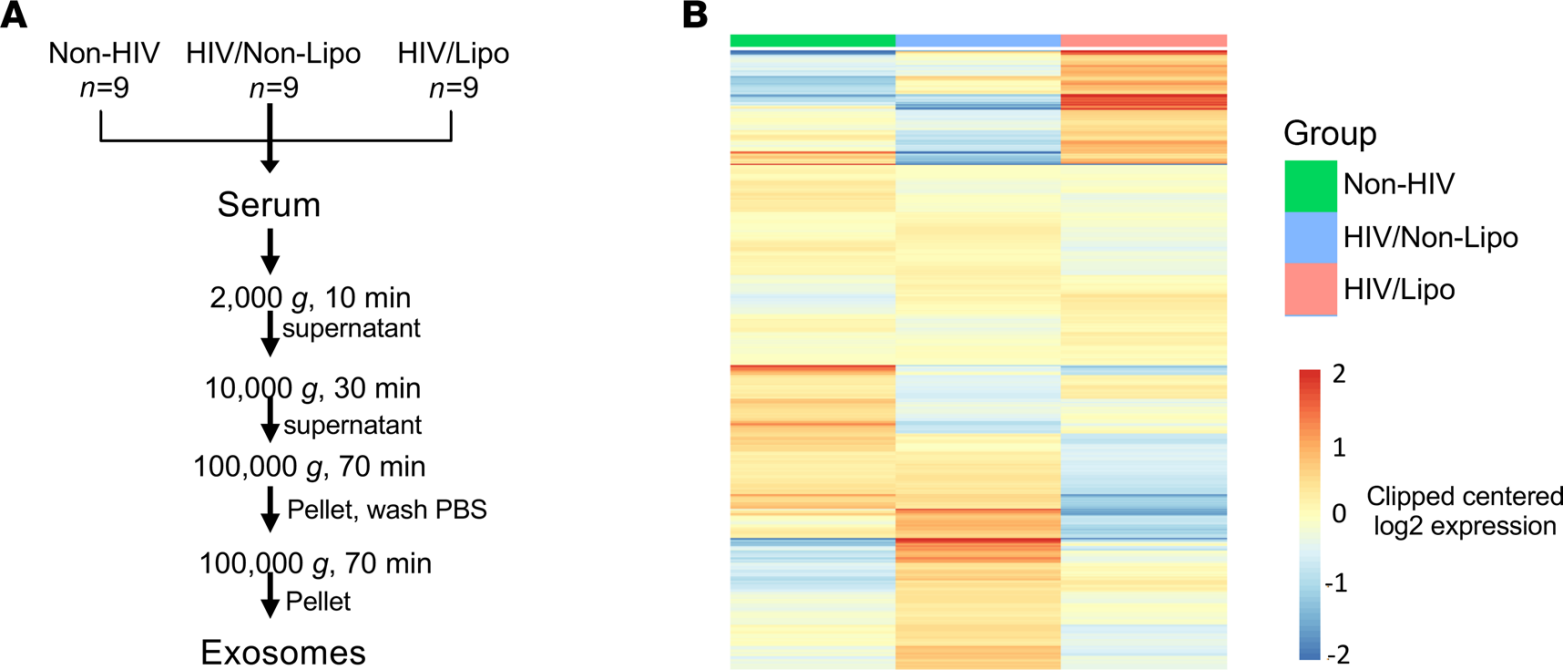
Isolation of circulating sEV miRNAs correlating to HIV infection and lipodystrophy. (**A**) Diagram representing the sEV isolation method. Exosomes were isolated from HIV/lipodystrophy, HIV/nonlipodystrophy, and non-HIV participants (*n* = 9 per group) by differential centrifugation protocol and subjected to qPCR-based miRNA profiling. (**B**) Heatmap showing the group average expression for each miRNA measured in serum. Red color indicates high expression; blue indicates low expression. Left column (non-HIV, indicated by green box at top); middle column (HIV nonlipo, indicated by blue box at top); right column (HIV lipo, indicated by pink box at top). *n* = 8–9 per group applies to all the panels in this figure.

**Figure 2:**
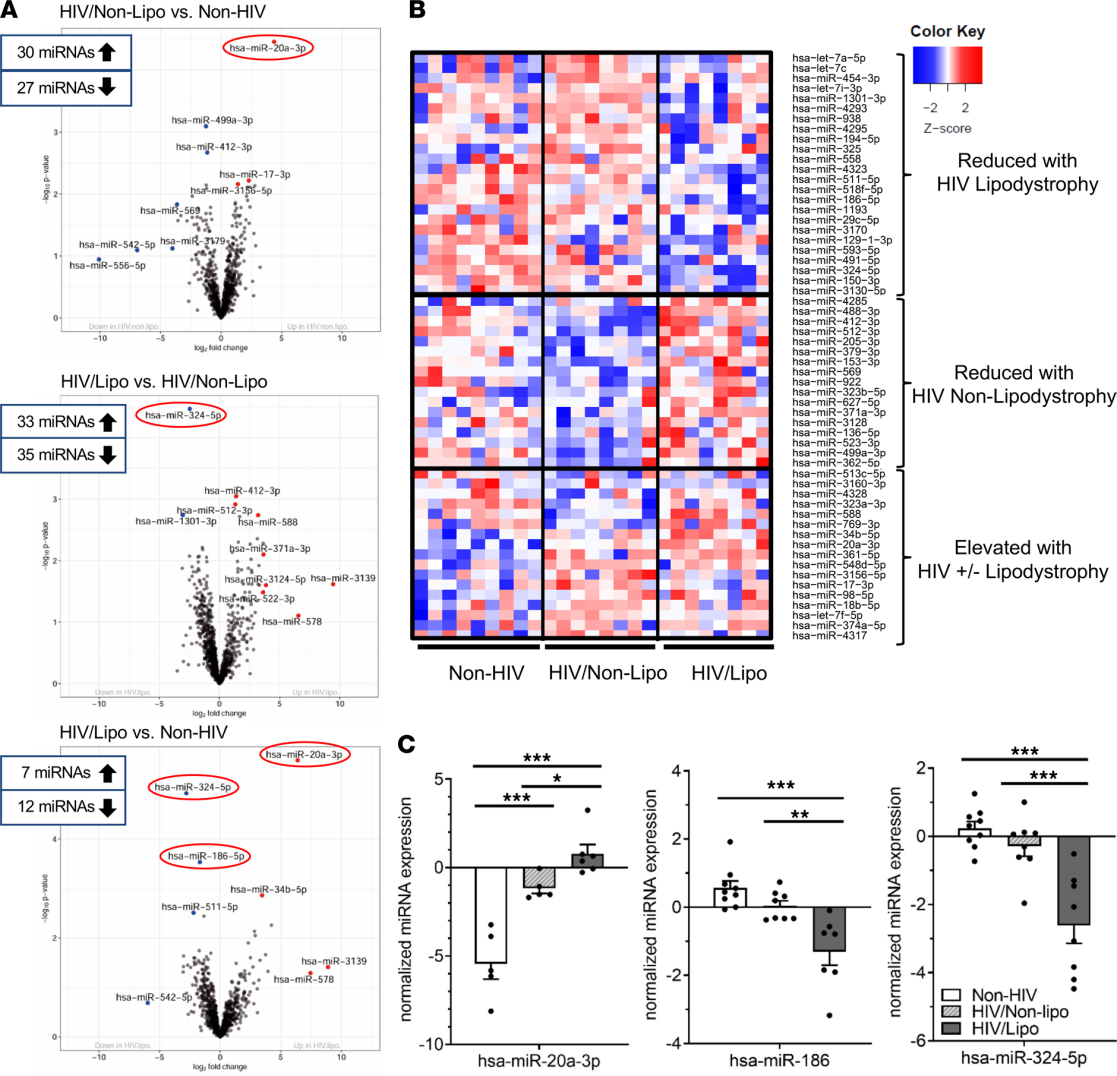
Circulating sEV miRNAs correlating to HIV infection and lipodystrophy. (**A**) Volcano plots representing the fold change and statistical significance for circulating sEV miRNAs for each of the group comparisons. The top regulated miRNAs are highlighted. The numbers in the rectangles indicate the number of upregulated and downregulated exosomal miRNAs (*P* < 0.05). The 3 miRNAs further studied (miR-20a-3p, miR-186, and miR-324-5p) are highlighted in a red circle. (**B**) Heatmap showing the abundance of the top regulated circulating sEV miRNAs shown in **A** in the conditions stated on the right side. High expression as indicated by normalized Ct is shown in red and low expression in blue. Each column represents an individual sample. (**C**) Normalized sEV miRNA expression obtained from miRNA profiling in each group for miR-20a-3p, miR-186, and miR-324-5p. Data are expressed as mean ± SEM. *P < 0.05, **P < 0.01, ***P < 0.001. *n* = 5–9 per group applies to all the panels in this figure; statistical comparison in **A**–**C** is assessed by Limma analysis.

As we have previously demonstrated, reduced adipose tissue *Dicer* in mice leads to changes in the expression levels of circulating sEV miRNAs (13). Given the reduction in adipose *Dicer* expression in HIV patients with lipodystrophy, we addressed whether circulating sEV miRNAs were also changed in these individuals. To do so, we isolated serum sEV using differential centrifugation protocol (15) from HIV/lipo, HIV/nonlipo, and non-HIV participants (Figure 1A). This protocol led to vesicles in the expected range for exosomes (50–200 nm), enriched in the sEV markers CD63 and TSG101, while depleted from the cellular marker calnexin (Supplemental Figure 1, A–C; supplemental material available online with this article; https://doi.org/10.1172/jci.insight.150399DS1). As shown in Figure 1B, each group (non-HIV, HIV/nonlipo, and HIV/lipo) displayed a distinct circulating sEV miRNA profile. Comparing the HIV/lipo and non-HIV participants, the levels of 7 sEV miRNAs were significantly upregulated while 12 were downregulated (Figure 2, A and B). In the comparison of HIV/nonlipo with non-HIV, 30 sEV miRNAs were significantly upregulated and 27 were downregulated (Figure 2, A and B). The miRNA profile of HIV participants with lipodystrophy was also compared with HIV participants without lipodystrophy. In this comparison, the expression of 33 miRNAs was significantly upregulated and 35 downregulated (Figure 2, A and B). Interestingly, although additional changes did not reach statistical significance, expression of many different miRNAs was generally decreased in the HIV/lipo group as observed by the left shift in the base of the volcano plots shown in Figure 2A and the bluish tone of the upper half column for HIV/lipo in the heatmap displayed in Figure 2B. This observation is in line with a previous observation from our group (13) and highlights that many of the differentially expressed miRNAs were downregulated. Thus, HIV infection either with or without lipodystrophy leads to significant changes in multiple circulating sEV miRNAs. Decreases in the circulating sEV miRNA population were also seen that specifically associated with lipodystrophy.

The top differentially regulated miRNAs are shown in Figure 2B and can be clustered in 3 major groups: sEV miRNAs reduced in HIV lipodystrophy, others reduced in HIV without clinical evidence of lipodystrophy, and others elevated in HIV regardless of a lipodystrophy presentation. Assessing for differences using a more stringent statistical approach (FDR < 0.05) revealed miR-20a-3p, miR-324-5p, and miR-186 as the top changed miRNAs. Notably, miR-20a-3p showed a stepwise progression and was significantly elevated in HIV/nonlipo compared with non-HIV and even further elevated in HIV/lipo (Figure 2C). In contrast, miR-324-5p and miR-186 were specifically downregulated in the HIV/lipo group compared with the other groups (Figure 2C). Among all participants, upregulation of miR-20a-3p correlated with downregulation of miR-324-5p (ρ = –0.71, *P* = 0.002) and miR-186 (ρ = –0.74, *P* = 0.002). In addition, downregulation of miR-324-5p correlated with downregulation of miR-186 (ρ = 0.54, *P* = 0.006) (Table 2). Interestingly, expression of these circulating miRNAs in sEVs correlated significantly to adipose *Dicer* expression among all participants (miR-20a-3p, ρ = –0.61, *P* = 0.01; miR-324-5p, ρ = 0.39, *P* = 0.05; miR-186, ρ = 0.53, *P* = 0.008) (Table 3) and also strongly correlated with dorsocervical adipose tissue area (our clinical measure of lipodystrophy) (Table 3). Levels of miR-20a-3p correlated with respiratory quotient (ρ = –0.64, *P* = 0.008), triglycerides (ρ = 0.52, *P* = 0.04), insulin (ρ = 0.67, *P* = 0.005), and HOMA-IR (ρ = 0.50, *P* = 0.047), while miR-186 related to reduced CD4^+^ cell count (ρ = 0.55, *P* = 0.04) (Table 3). Taken together, these data suggest that the alteration in the levels of circulating sEV-carried miRNAs, i.e., miR-20a-3p, miR-186, and miR-324-5p, might serve as markers for the degree of lipodystrophy among individuals with HIV.

### Altered pattern of circulating sEV-carried miRNAs in HIV lipodystrophy is concordant with changes in aDicer-KO mice and transfection with Dicer siRNA.

**Figure 3:**
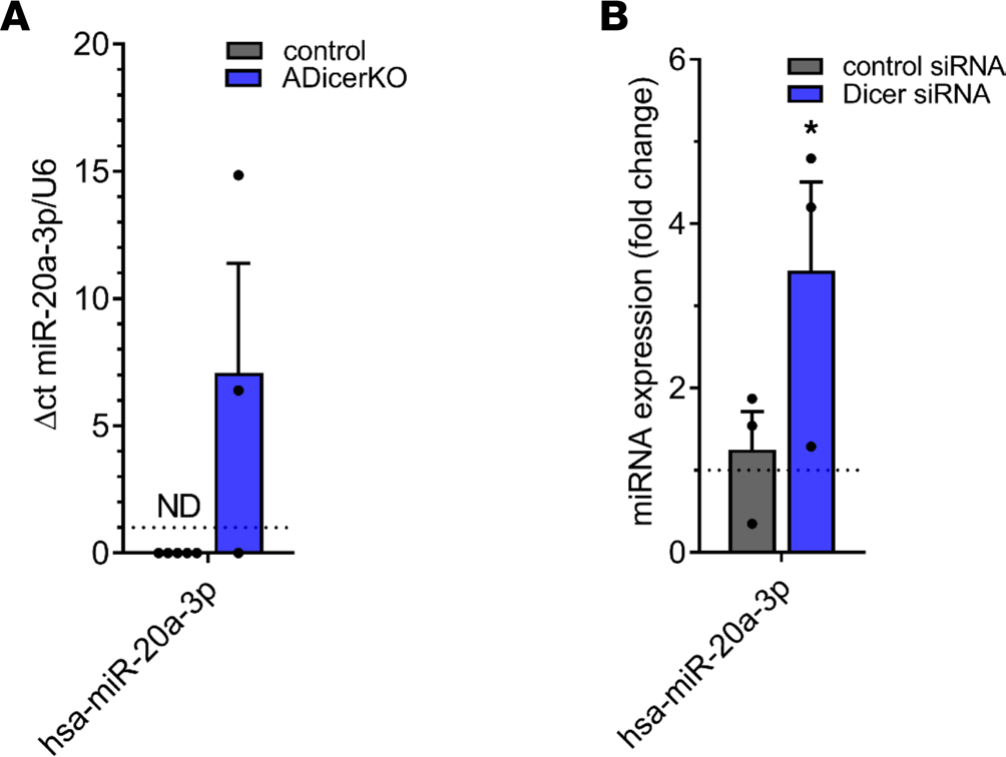
Expression of miR-20a-3p in alternative models of reduced *Dicer* expression. (**A**) Normalized sEV miRNA expression for miR-20a-3p in a*Dicer*-KO mice versus control. (**B**) Normalized sEV miRNA expression for miR-20a-3p in adipocytes transfected with *Dicer* siRNA versus control. Data are expressed as mean ± SEM. *P < 0.05. *n* = 5 sera pools for control mouse group (each pool derives from sera of 3 mice), *n* = 3 sera pools for a*Dicer*-KO mouse group (each pool derives from sera of 3 mice), *n* = 3 per group applies for **B**. ND, not detected.

sEVs were isolated from the pooled serum — each sample corresponds to the serum of 3 mice, mixed female and male (2:1) control mice (Adiponectin-Cre-*Dicer*^fl/fl^) and a*Dicer*-KO mice (10–16 weeks old) — by standard ultracentrifugation protocol and subjected to RNA isolation. qPCR revealed miR-20a-3p was detectable in 2 of the 3 a*Dicer*-KO mice and none of the 5 control mice (Figure 3A). We have previously reported on reduced miR-186 and miR-324-5p in a*Dicer*-KO mice (13). In addition, we have now performed silencing transfection of preadipocytes with *Dicer* siRNA. miR-20a-3p was significantly upregulated compared with control siRNA (Figure 3B). Herein, we show 3 models in which miR-20a-3p is upregulated, including in the circulation of humans with HIV lipodystrophy and reduced subcutaneous adipose *Dicer* expression, in human preadipocyte cell culture models with transfection of *Dicer* siRNA in knockdown studies, and in the circulation of a murine model with adipose *Dicer* knockout.

### Gene expression signatures of adipocyte differentiation decreased by transfection of miR-20a-3p or the anti-mers of miR-186 and miR-324-5p.

**Figure 4:**
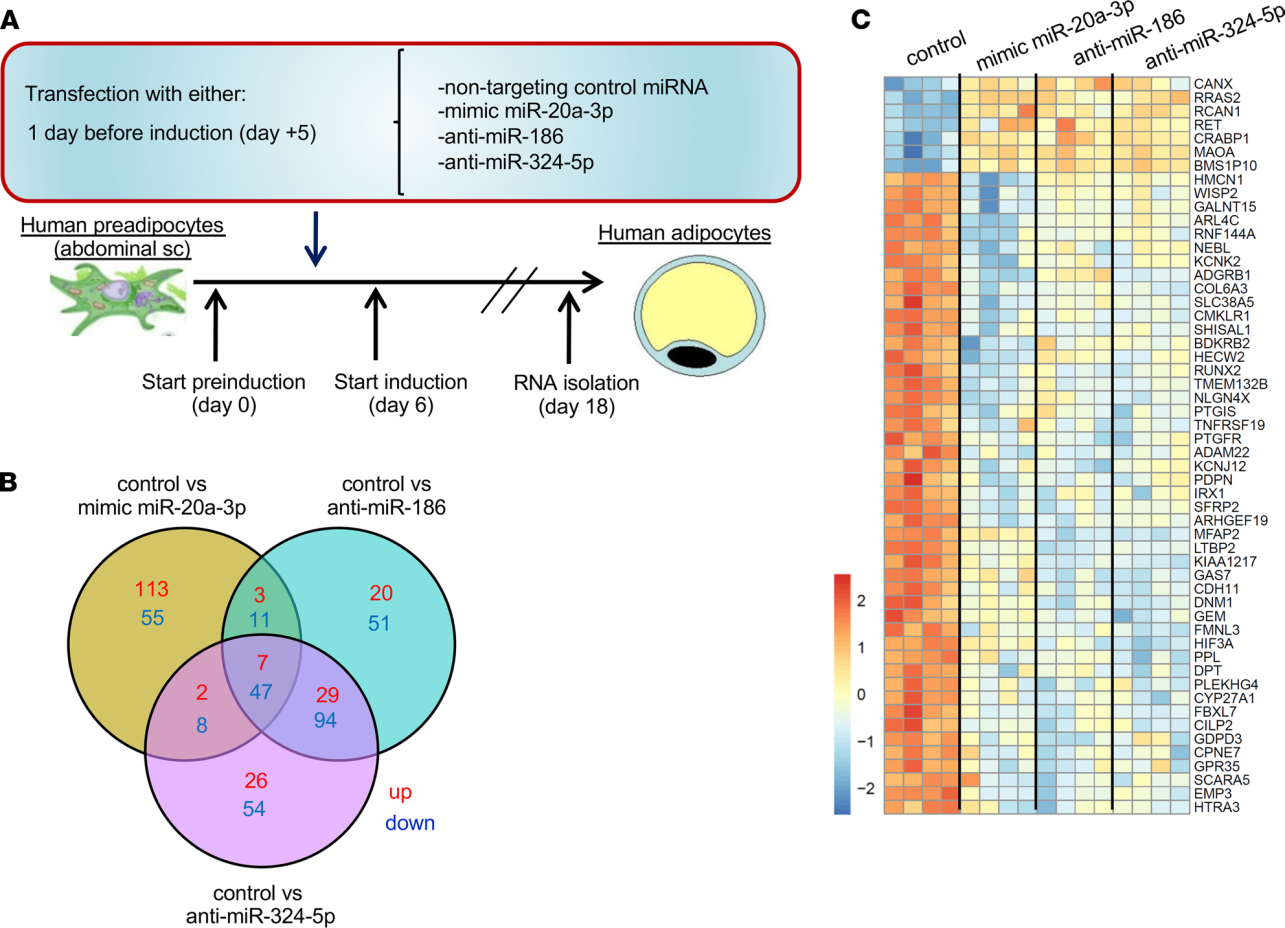
Overexpression of miR-20a-3p and inhibition of miR-186 and miR-324-5p alter transcriptome of differentiating adipocytes. (**A**) Diagram representing the experimental setup. (**B**) Venn diagram showing the number of genes upregulated (red) and downregulated (blue) for each comparison and the overlap among them. (**C**) Heatmap showing the commonly regulated genes by all treatments from the center of the Venn diagram in **B**. Red indicates high expression; blue indicates low expression. *n* = 4 per group in all the panels; statistical comparisons in **B** and **C** assessed by Limma analysis.

Given these previous observations, we assessed whether the increased levels of sEV-carried miR-20a-3p or decreased levels of sEV-carried miR-186 and miR-324-5p might underlie the metabolic alterations observed in HIV lipodystrophy, specifically with respect to adipocyte dysregulation in the white adipose depot, including altered patterns of adipocyte differentiation capacity. To address this question, human preadipocytes were transfected with miR-20a-3p mimic, anti–miR-186, anti–miR-324-5p, or control nontargeting miRNA and then cultured in differentiation medium for 12 additional days to give rise to mature adipocytes, after which RNA was collected and subjected to RNA-Seq (Figure 4A). The transfection was performed in parallel with a fluorescence-labeled miRNA or a positive control miRNA targeting aldolase-A to confirm success. Analysis of the RNA-Seq data revealed 125 genes significantly upregulated, and 121 genes significantly downregulated in the mimic miR-20a-3p–treated cells, compared with miRNA control–treated cells (Figure 4B). Fifty-nine genes were significantly upregulated, and 203 genes were significantly downregulated when treating the cells with anti–miR-186 versus control group, and 64 genes were significantly upregulated and 203 miRNAs significantly downregulated when treating the cells with anti–miR-324-5p (Figure 4B). Surprisingly, both anti-miRs highly overlapped in their gene expression pattern while mimic miR-20a-3p resulted in a more distinct pattern (Figure 4C and Supplemental Figure 2, A and B). Among the pathways upregulated by mimic miR-20a-3p, we found several related to cellular adhesion and activity of the actin cytoskeleton (FAK-adhesion, ECM-receptor interaction, regulation of actin cytoskeleton) and others related to inflammatory processes (cytokine-cytokine receptor activation, hematopoietic cell lineage) (Supplemental Figure 2C). Only the proteoglycan pathway was slightly downregulated by mimic miR-20a-3p (Supplemental Figure 2C). In contrast, both anti-miRs upregulated pathways related to catabolism, such as oxidative phosphorylation, TCA cycle, or fatty acid degradation, and downregulated others related to cell adhesion and ligand pathways, such as Hippo and Wnt signaling pathways (Supplemental Figure 2D).

**Figure 5:**
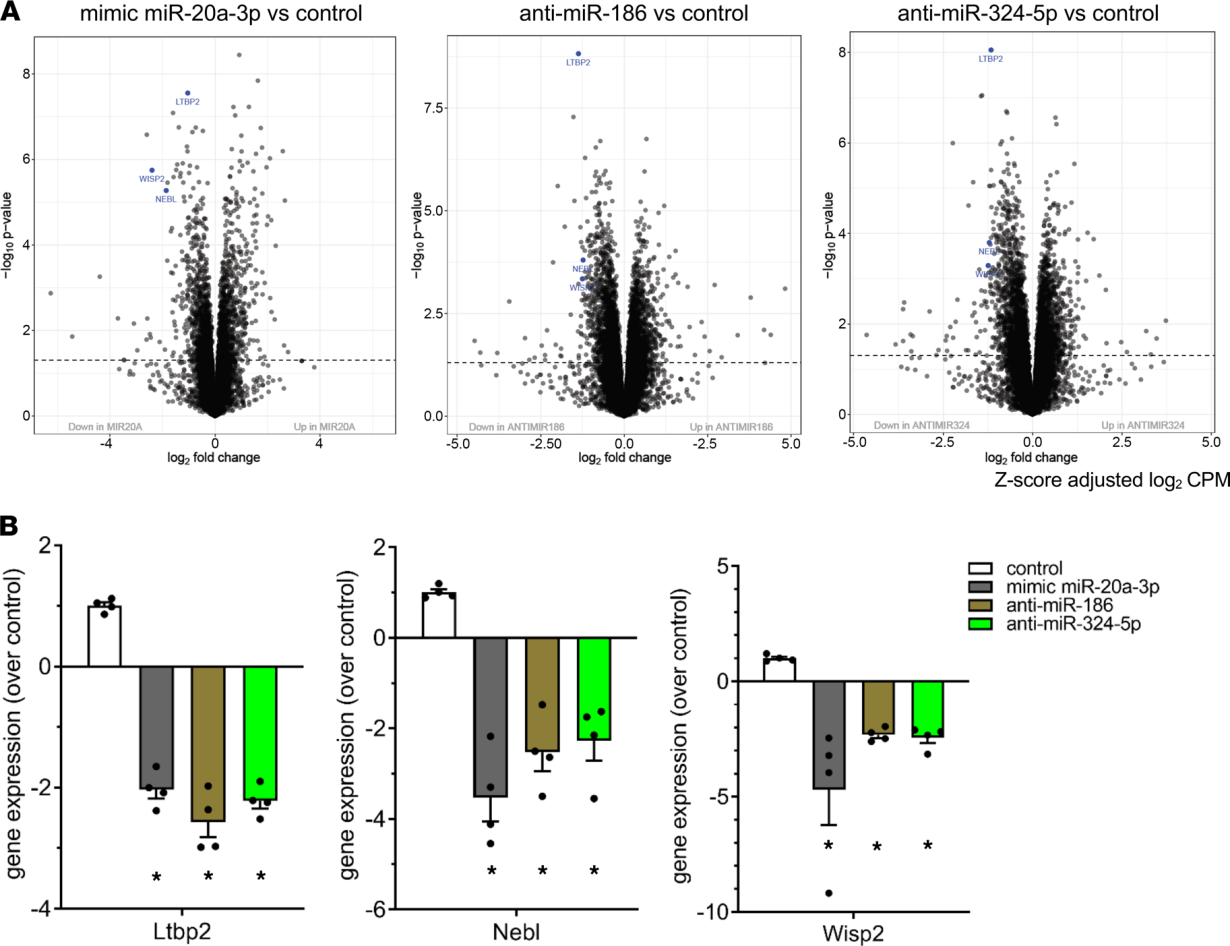
Overexpression of miR-20a-3p and inhibition of miR-186 and miR-324-5p of differentiating adipocytes demonstrates downregulation of *Ltbp2*, *Nebl*, and *Wisp2*. (**A**) Volcano plots illustrating the fold change and statistical significance for the profile of each comparison. The dashed line in each plot indicates *P* value 0.05. The genes that are the subject of further study (*Ltbp2*, *Nebl*, and *Wisp2*) are highlighted in blue. (**B**) Bar graphs showing the degree of downregulation of *Ltbp2* (left), *Nebl* (middle), and *Wisp2* (right graph) induced by each of the treatments. Data are expressed as mean ± SEM. *P < 0.05. *n* = 4 per group in all the panels; statistical comparisons in **A** and **B** assessed by Limma analysis.

To identify genes that might be coordinately regulated by the upregulation of circulating sEV-carried miR-20a-3p and the downregulation of miR-186 and miR-324-5p, we sought genes that would be regulated by all these 3, the mimic miR-20a-3p, anti–miR-186, and anti–miR-324-5p, in the same direction. This analysis revealed that 7 genes were similarly upregulated and 47 genes were similarly downregulated by these treatments (Figure 4B). The commonly regulated genes by all treatments are shown in a heatmap (Figure 4C). The gene with the most significant downregulation by all 3 treatments was latent transforming growth factor beta binding protein 2 (*Ltbp2*) as shown by volcano plots (Figure 5A) and bar graphs (Figure 5B). In addition, other genes very significantly downregulated by all 3 treatments included nebulette (*Nebl*) and WNT1-inducible-signaling pathway protein 2 (*Wisp2*) (Figure 5, A and B). *Ltbp2* is thought to relate to SREBP-1b and SREBP-1c and was shown to associate with BMI-adjusted waist circumference in GWAS (16). Interestingly, *Nebl* is a predicted target of all 3 miRNAs (miR-20a-3p, miR-324-5p, and miR-186) that was identified using several miRNA target prediction tools (TargetScan and Diana databases, target score > 85%). *Wisp2* participates in the induction of brown adipose tissue function, augmentation of insulin sensitivity, and regulation of preadipocyte commitment and PPARγ activation (17–19). Thus, our data suggest that the increased levels of circulating sEV-carried miR-20a-3p and decreased miR-186 and miR-324-5p associated with HIV lipodystrophy might influence adipocyte differentiation in several key ways, by affecting pathways related to lipid catabolism, inflammation, or cellular adhesion, among others, through effects on *Ltbp2*, *Nebl*, and *Wisp2*.

**Figure 6:**
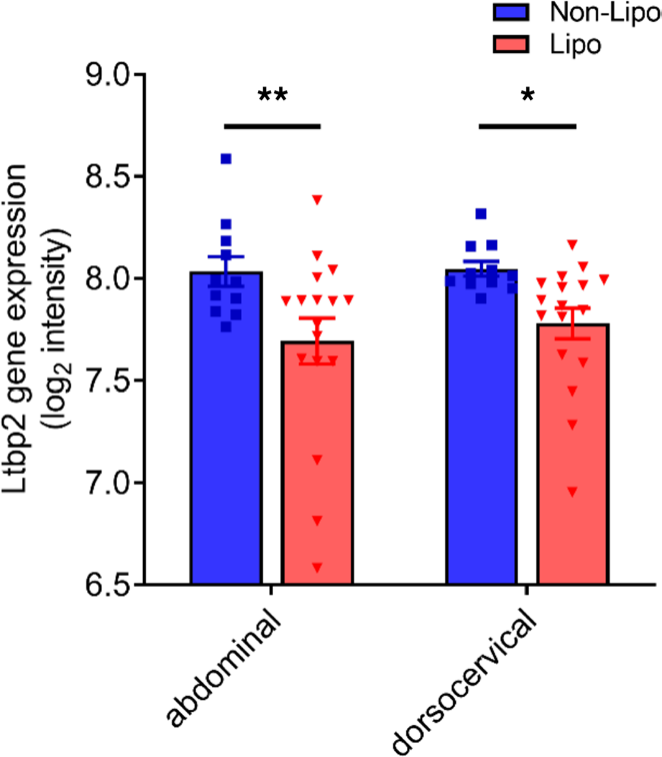
*Ltbp2* expression is downregulated in another cohort of HIV-infected patients with lipodystrophy. *Ltbp2* gene expression in the abdominal subcutaneous and dorsocervical adipose tissue depots from a cohort of HIV-infected patients without or with lipodystrophy. *n* = 11 for nonlipodystrophic patients, *n* = 17 for lipodystrophic patients. Data are expressed as mean ± SEM. *P < 0.05, **P < 0.01; statistical comparisons were assessed by Limma analysis.

In addition, utilizing the publicly available National Center for Biotechnology Information Gene Expression Omnibus database GSE28073 comparing gene expression in HIV-infected patients (20), we found a reduction of *Ltbp2* in both abdominal and dorsocervical adipose tissues from patients with HIV lipodystrophy compared with patients with HIV without lipodystrophy (Figure 6).

### Assessment of adipocyte differentiation and inflammatory phenotype in knockdown studies of transfected human adipocytes.

**Figure 7:**
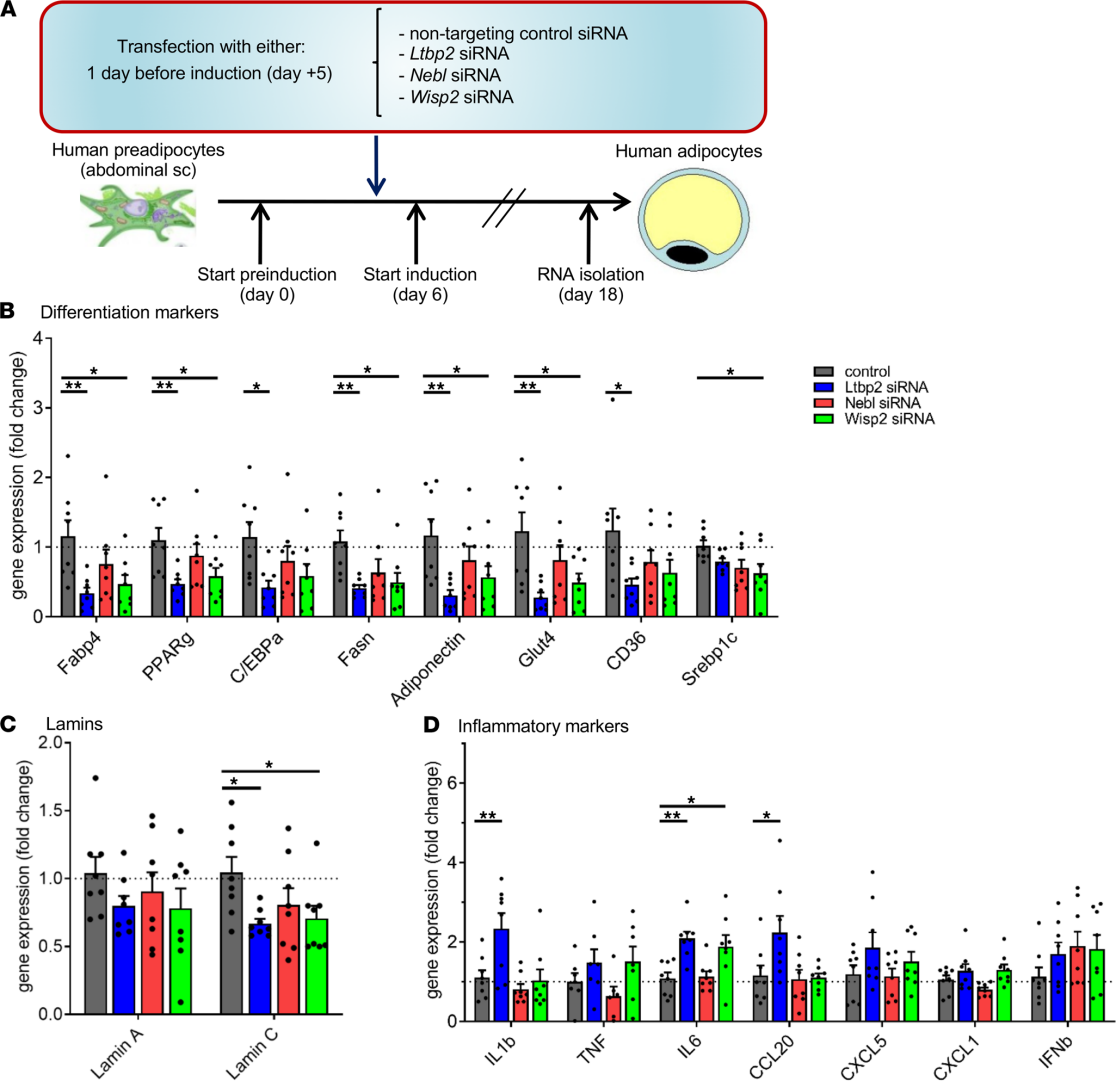
*Ltbp2* regulates adipocyte differentiation, *Lamin C* expression, and inflammation. (**A**) Diagram representing the experimental setup. (**B**) Effect of the treatment of preadipocytes with negative control, *Ltbp2*, *Nebl*, and *Wisp2* siRNA on the indicated differentiation markers. (**C**) Effects of the indicated treatments on the expression of *Lamin A* and *Lamin* *C*. (**D**) Effects of the indicated treatments on the expression of the inflammatory markers shown below. Data are expressed as mean ± SEM. *P < 0.05, **P < 0.01. *n* = 8 in all panels; statistical comparisons in **B**–**D** were performed by 1-way ANOVA followed by Dunnett’s test comparing individual groups to control.

In order to determine the effects of the downregulation of *Ltbp2*, *Nebl*, and *Wisp2* and their potential synergy in adipocyte differentiation, we performed silencing transfection of preadipocytes with either *Ltbp2* siRNA, *Nebl* siRNA, or *Wisp2* siRNA individually, or a cocktail of all 3 siRNAs (containing one-third of each siRNA dose) 1 day prior to induction of differentiation. As a control, a nontargeting siRNA was used in parallel. RNA was collected at the end of the differentiation and subjected to RNA-Seq (Figure 7A). As shown in Supplemental Figure 3, single siRNA treatment efficiently downregulated (>80% reduction) the target genes. Triple siRNA combination efficiently downregulated *Ltbp2* (~80% reduction) but had a weaker but still significant reduction on *Nebl* (~50% reduction) and had a very modest nonsignificant effect on *Wisp2* (~20% reduction) (Supplemental Figure 3). As the triple siRNA combination had one-third of the dose of each siRNA, this differential targeting capacity for each target is likely due to lower efficiency at slightly lower doses.

**Figure 8:**
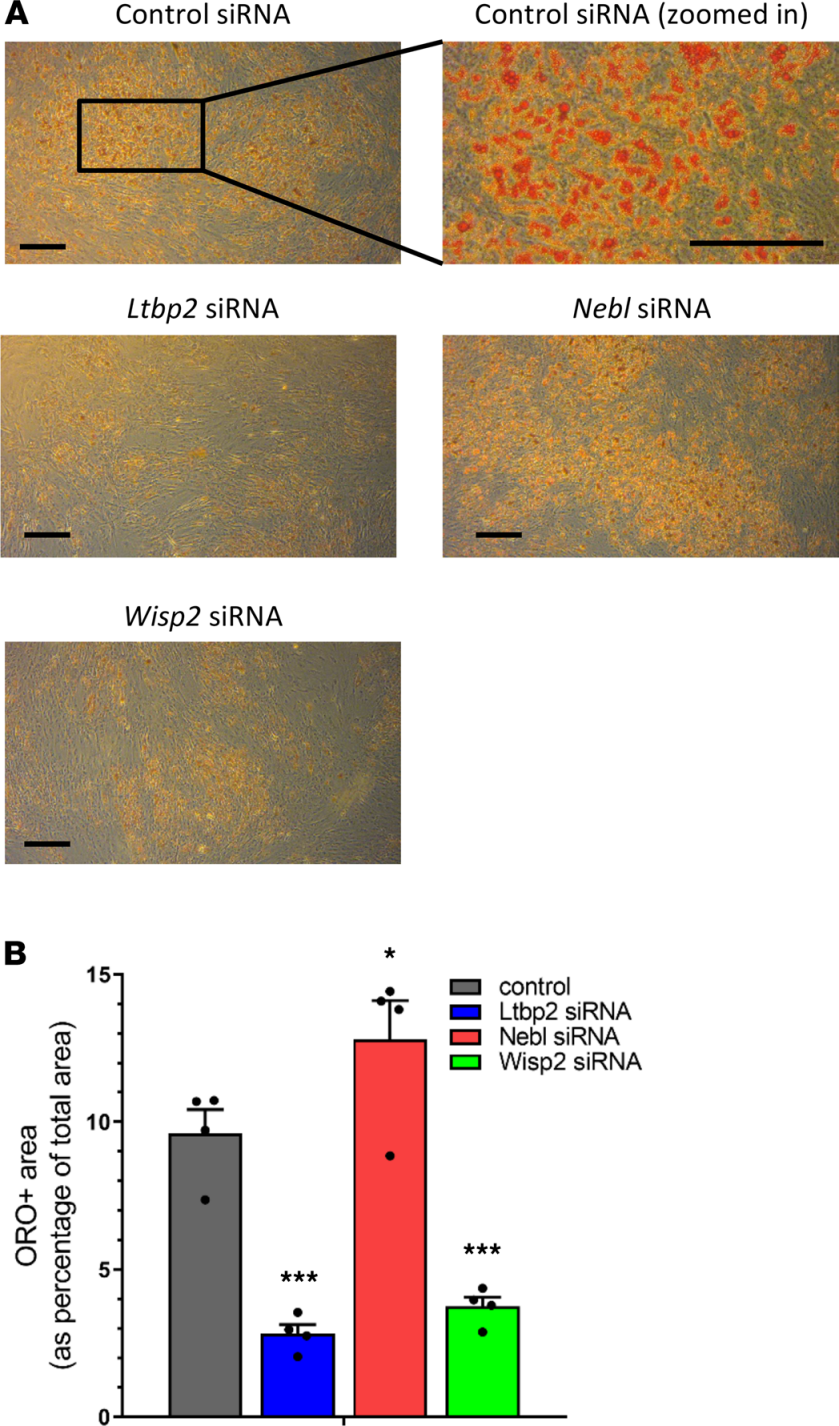
Knockdown of *Ltbp2* demonstrates reduced adipocyte differentiation. (**A**) Histologic images of adipocytes treated with *Ltbp2*, *Nebl*, and *Wisp2* siRNA stained with oil red O. (**B**) Quantitative measure of oil red O staining as a percentage of total area. Scale bar: 100 μm. *n* = 4, data are expressed as mean ± SEM. *P < 0.05, ***P < 0.001; statistical comparisons was performed by 1-way ANOVA followed by Dunnett’s test comparing individual groups to control.

We next analyzed how the downregulation of *Ltbp2*, *Nebl*, and *Wisp2* might affect processes involved in lipodystrophy, such as adipocyte differentiation. We found that knockdown of *Ltbp2* significantly downregulated multiple gene markers of adipocyte differentiation, such as *Fabp4*, *Ppar*γ, *C/ebpa*, *Fasn*, *adiponectin*, *Glut4*, and *CD36* (Figure 7B). Knocking down *Wisp2* also led to a general decrease of several of these adipocyte differentiation markers, with effects on *Fabp4*, *Ppar*γ, *Fasn*, *adiponectin*, *Glut4*, and *Srebp1c* reaching statistical significance (Figure 7B). In contrast, *Nebl* siRNA did not lead to any significant changes. Combined knockdown of these targets using a combination of *Ltbp2* siRNA, *Nebl* siRNA, and *Wisp2* siRNA was additionally assessed in a supportive analysis and resulted in a significant downregulation of *Fabp4*, *Ppar*γ, *C/ebpa*, *Fasn*, *adiponectin*, *Glut4*, and *CD36* (Supplemental Figure 4B). Downregulation of genes related to adipocyte differentiation were generally lower with *Ltbp2* siRNA in contrast with the triple-combined siRNA (but at one-third of the concentration), suggesting the effect was driven predominantly by *Ltbp2* siRNA. Mutations in lamins A and C have been associated with lipodystrophy and metabolic complications (21–23). In our studies, compared with the control siRNA, there was a significant downregulation of *Lamin C* expression in both *Ltbp2* siRNA and *Wisp2* siRNA groups, while *Lamin A* expression did not appear to differ among all groups (Figure 7C and Supplemental Figure 4C). We also demonstrate decreased adipocyte differentiation in histological studies utilizing oil red O staining. Histologic images and quantitative data showed reduced oil red O staining after adipocytes were transfected with siRNA for *Ltbp2* and *Wisp2* when compared with the control (Figure 8 and Supplemental Figure 5).

**Figure 9:**
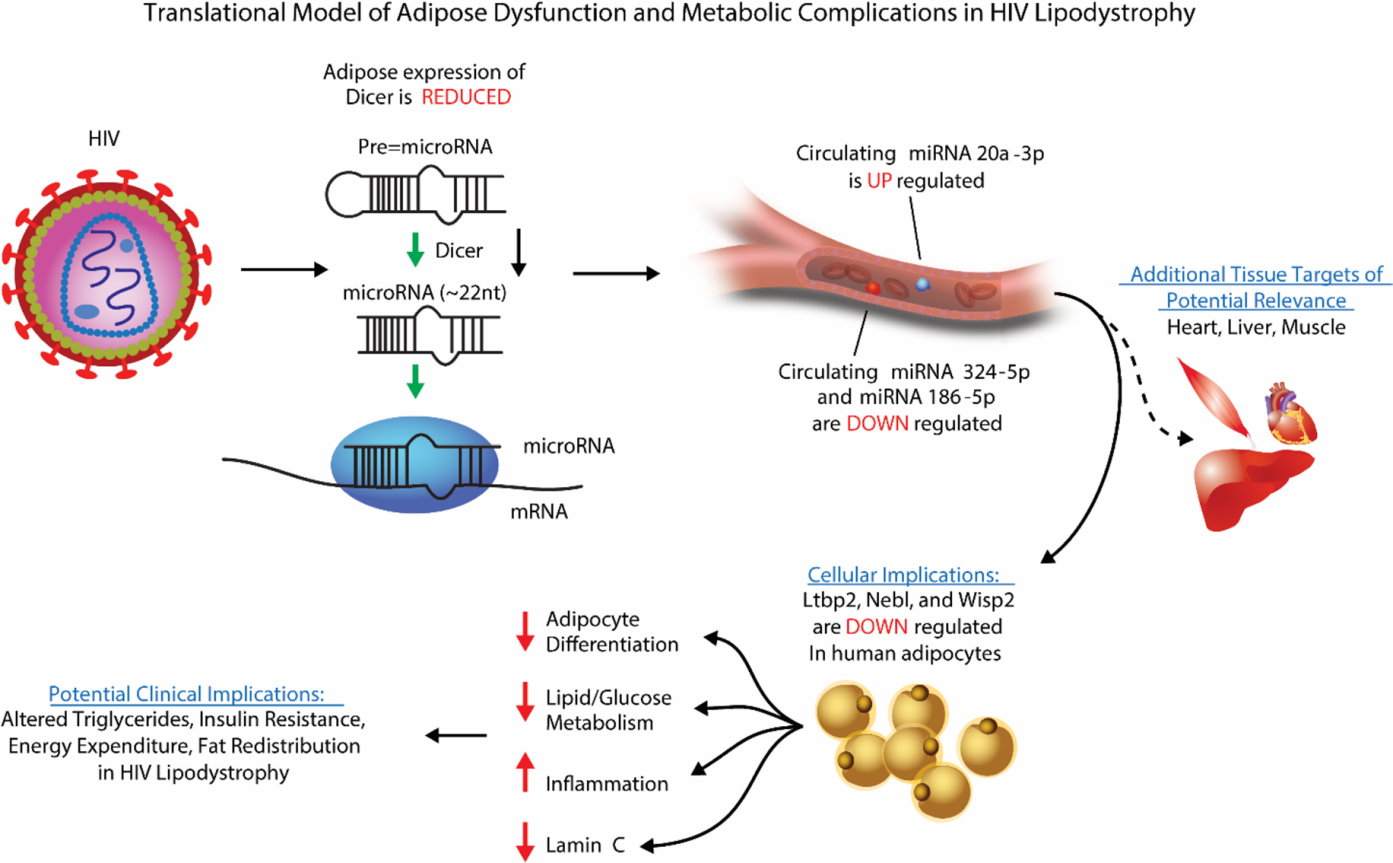
Hypothesized translational model of adipose dysfunction and metabolic complications in HIV lipodystrophy. Accessory proteins of the HIV may suppress *Dicer* expression in the adipose tissue. Lack of *Dicer* in adipose tissue leads to lipodystrophic changes in fat redistribution, metabolic dysregulation, and alteration of the levels of circulating exosomal miRNAs; i.e., sEV-carried miR-20a-3p is upregulated, and miR-324-5p and miR-186 are downregulated in serum taken from lipodystrophic patients with HIV. These sEVs would target preadipocytes, inducing downregulation of *Ltbp2*, *Nebl*, and *Wisp2*, which would subsequently impair adipocyte differentiation, inflammation, and *Lamin C* expression. Thus, reduced dicer expression in the adipose among HIV-infected patients may contribute to an altered miRNA signature and adipocyte differentiation and inflammation, which could have potential clinical implications relevant to HIV lipodystrophy. Other tissues affected in HIV lipodystrophy, such as the heart, liver, and muscle, could also be relevant targets of this pathway.

Another process intimately related to lipodystrophy is the activation of inflammatory pathways. Again, *Ltbp2* knockdown had the greatest effects, leading to upregulation of *IL1b*, *IL6*, and *Ccl20*, while the other treatments had no or very minor effects (Figure 7D and Supplemental Figure 4D). We saw consistent but nonsignificant trends toward increased expression of *IFNβ* in response to knockdown of *Ltbp2*, *Nebl*, and *Wisp2*. Thus, our data suggest that *Ltbp2* is important for proper adipocyte differentiation and control of inflammation. The downregulation of *Ltbp2* induced by elevated sEV-carried miR-20a-3p or decreased sEV miR-186 and miR-324-5p in patients with HIV lipodystrophy may underlie the impairment of adipose tissue development/differentiation and the increased activation of inflammatory processes seen in such patients and could be a potential target for future clinical studies (Figure 9).

No effects of knockdown of *Ltbp2*, *Nebl*, or *Wisp2* were seen on markers of mitochondrial function, *Tomm20* and *mtND2*. When assessing effects on markers of oxidative stress, no effects were seen on *Catalase*, *Sod1*, *Sod2*, or *NADPHox-p40phos*, but *Hmox1* was decreased significantly in response to *Ltbp2* (Supplemental Figure 6).

In exploratory analyses of effects on brown fat gene expression, we applied a beiging differentiation protocol to the preadipocytes. We observed nonsignificant decreases in brown adipose marker *Ucp1* with *Ltbp2*, *Nebl*, and *Wisp2* siRNA compared with the control. In addition, *Prdm16*, a key beige adipose marker, demonstrated a relative decrease following transfection of *Ltbp2*, *Nebl*, and *Wisp2* siRNA when compared with the control (Supplemental Figure 7).

## Discussion

The HIV population frequently presents with a redistribution of fat and metabolic abnormalities, including dorsocervical adipose lipohypertrophy, ectopic fat accumulation, subcutaneous adipose dysfunction, insulin resistance, and dyslipidemia. These features closely resemble the phenotype of a*Dicer*-KO mice (8), surmising a role for adipose-derived miRNAs in the pathogenesis of this syndrome. Indeed, downregulation of *Dicer* expression in fat and subsequent dysregulation of adipose-derived miRNAs, many of which may be secreted in sEV and function as adipokines (13), could provide a novel mechanism for altered adipose function and related metabolic abnormalities in HIV lipodystrophy. Interestingly, HIV-related accessory proteins, for example viral protein R and trans-activator of transcription, have been reported to suppress *Dicer* expression (6, 24, 25). While suppression of *Dicer* may have evolved as a mechanism to enhance infectivity of the HIV, there may be unintended metabolic consequences leading to a lipodystrophic phenotype.

To begin to investigate the role of *Dicer* in adipose and metabolic regulation in HIV, we previously assessed subcutaneous adipose expression of *Dicer* in a group with well-defined lipodystrophy and demonstrated reductions in adipose *Dicer* expression in association with key brown fat genes and clinical parameters (6). In the current study, we have performed sEV miRNA profiling in this well-phenotyped cohort of individuals with and without HIV in whom we have previously characterized adipose expression of *Dicer*. We hypothesized a unique miRNA signature among those with HIV lipodystrophy in relationship to dysregulation of *Dicer*. In these analyses evaluating sEV miRNA profiles, we identified 3 specific miRNAs (miR-20a-3p, -186, and -324-3p) as the top differentially regulated miRNAs using a stringent statistical approach. Notably, these miRNAs demonstrated a unique signature strongly related to *Dicer* expression and well-defined clinical lipodystrophy characteristics, including increased dorsocervical adipose tissue, as well as key metabolic and immune indices. In support of our findings, we were able to demonstrate upregulation of miR-20a-3p across 3 adipose-specific models of reduced *Dicer* expression: (a) in the circulation among patients with HIV lipodystrophy and reduced *Dicer* expression in the abdominal subcutaneous depot, (b) in the circulation of the a*Dicer*-KO mouse, and (c) in a preadipocyte culture model transfected with *Dicer* siRNA. Follow-up experiments to manipulate these identified miRNA pathways in human cultured preadipocytes led to identification of potentially novel corresponding adipose gene targets of these miRNAs. Subsequent knockdown experiments of these genes through silencing transfection studies demonstrated key metabolic roles relevant to glucose and lipid metabolism.

We studied an HIV population who were well treated on ART, a group representative of the large population of aging individuals who are living with chronic HIV infection and demonstrate good immunologic control but nonetheless have an increased risk for mortality driven in part by metabolic complications. The HIV lipodystrophy cohort was identified based on the presence of dorsocervical lipohypertrophy on physical exam and confirmed based on quantitative measurements on MRI. Moreover, the cohort demonstrated reduced extremity fat via DXA, suggesting the common phenotype of loss of subcutaneous fat. *Dicer* expression was lowest in those with imaging-confirmed lipodystrophy, followed by HIV without lipodystrophy and then non-HIV controls (6).

We have previously shown the dorsocervical adipose tissue is a beige fat–like adipose depot that may develop as a compensatory mechanism to lipodystrophy (26). In a prior study we showed that reduced *Dicer* expression in subcutaneous adipose tissue is related inversely to dorsocervical adipose tissue accumulation. We now demonstrate that increased dorsocervical adipose tissue is strongly correlated to the miRNA signature we have identified, such that greater dorsocervical adipose tissue is associated with upregulation of miR-20a-3p and downregulation of miR-186 and miR-324-5p. Moreover, in exploratory analyses, we observed potential effects of key dysregulated genes in this study, *Ltbp2*, *Nebl*, and *Wisp2*, on 2 genes involved in browning of white adipocytes, *Ucp1* and *Prdm16*. Further studies should assess the effects of dicer-related downstream effect on brown and beige fat pathways in specific adipose depots.

In our study, pathway analyses revealed that highly relevant clusters important to metabolism and cytokine signaling were significantly affected by miR-20a-3p mimic as well as anti–miR-186 and anti–miR-324. These additional analyses provide supporting evidence that the 3 top differentially regulated miRNAs identified in this study are linked to adipocyte biology and physiologic systems applicable to HIV lipodystrophy. In addition, there may be clinical utility to evaluating whether these 3 sEV miRNAs could serve as a diagnostic tool using a simple, noninvasive technique to identify a clinical presentation of HIV lipodystrophy, for which objective measures may be lacking. Moreover, we saw that upregulation of miR-20a-3p related to greater triglycerides and insulin resistance, further suggesting a link to the clinical lipodystrophy phenotype. Further studies may identify other miRNAs potentially important in mediating metabolic effects in relationship to reduced *Dicer* in HIV.

There are limited adipose and metabolic data about the roles of the 3 top differentially regulated miRNAs identified in this study in other cellular and disease models. A recent study of *Mycobacterium tuberculosis* suggested that overexpression of miR-20a-3p contributed to mycobacterial survival in a macrophage cell line and may aid in the host immune response (27). In this way, changes in miR-20a-3p and other miRNAs could help enhance survival of HIV in the host and have a secondary consequence of metabolic complications. In addition, miR-20a-3p has also been implicated in the MAPK signaling pathway and phosphatidylinositol signaling system (28). Consistent with the current study, miRNA-20a-3p has been shown to be elevated among patients with type 2 diabetes mellitus compared with healthy individuals when isolated from circulating plasma ectosomes (29). A recent study showed that miR-186 is upregulated in a T lymphoblast cell line following acute HIV infection, and this is associated with reduced *Dicer* expression in these cells (30). In our study, we have shown, in contrast, reduced miR-186 in circulating sEVs from chronically treated HIV-infected patients on ART, demonstrating reduced *Dicer* expression in the adipose depot. With regard to miR-324-5p, which we show to be downregulated in HIV lipodystrophy, this miRNA has also been shown to be downregulated in circulating sEVs of patients with type 2 diabetes mellitus while demonstrating an inhibitory effect on diabetes-related inflammation (31). miR-324-5p overexpression has been linked to enhanced glucose uptake in oxygen glucose–deprived neurons (32) and has been reported to promote Wnt signaling (33). Consistent with this, we found downregulated Wnt signaling in adipocytes following treatment with anti–miR-324-5p.

In the context of HIV lipodystrophy, few other miRNAs have been reported to have a potential role. One study demonstrated that miR-218 was upregulated in the subcutaneous adipose of patients with HIV lipodystrophy, and this was associated with reduced lipin1 levels, a putative target of this miRNA. Furthermore, cotreatment of 3T3-L1 cells with a miR-218 mimic and lopinavir/ritonavir decreased *Glut4* levels (34, 35). This prior study also reported that 21 miRNAs out of 754 were overexpressed by 2-fold in the subcutaneous adipose among 8 individuals with HIV lipodystrophy compared with 8 uninfected individuals. Interestingly, in contrast to our findings in sEV miRNAs, the authors found that miR-186 was upregulated in the adipose depot but did not report any data on miR-20a-3p or miR-324-5p in the adipose tissue or on altered pattern of circulating miRNA.

We have previously reported significant miRNAs affected in the a*Dicer*-KO model (8) and now show general concordance between findings in these mouse and human studies, for example, in the relative increase in miR-20a-3p and reductions in reduced miR-186 and miR-324-5p. Some differences between studies could be due to selective sorting of certain miRNAs into sEVs (36, 37), differing biology in the mouse versus human, or differences in the mechanism of *Dicer* dysregulation in these models, e.g., adipose-specific total genetic knockout of Dicer in the mouse study (8) versus physiological regulation in multiple tissues by HIV-related factors in the current study. Taken together, these data from multiple experiments, including the observation of increased miR-20a-3p expression in preadipocytes in response to *Dicer* siRNA, suggest an effect of reduced dicer to increase miR-20a-3p and affect other miRNAs.

One interesting aspect of these studies was the finding that *Ltbp2* transcription was significantly downregulated in human preadipocyte cultures by all 3 miRNAs observed to be altered in HIV lipodystrophy. Similarly, expression of *Nebl* and *Wisp2* was also decreased by all 3 miRNAs. Furthermore, we showed that *Ltbp2* was reduced in both abdominal and dorsocervical adipose tissue biopsies from another cohort of patients with HIV who developed lipodystrophy when compared with adipose tissue from patients with HIV who did not develop lipodystrophy (20), further indicating a critical role of *Ltbp2* in HIV lipodystrophy. Moreover, in the present study, we demonstrated that downregulation of *Ltbp2* in adipocytes by siRNA affected key regulators of adipocyte differentiation, metabolic regulation, cellular structure, and inflammation. Indeed, knockdown of *Ltbp2* in human cell culture of preadipocytes consistently resulted in downregulation of several key genes associated with maturation of adipocytes, including *C/ebpa* and *CD36*, highlighting the role of *Ltbp2* as a potential mechanistic factor contributing to subcutaneous adipose dysfunction and related metabolic abnormalities in HIV lipodystrophy. To complement these changes in gene expression, we also found reduced oil red O expression in adipocytes transfected with *Ltbp2*. Of note, prior work in HIV has shown adipose dysfunction among individuals with HIV, with lower mRNA concentrations of key adipogenic differentiation factors, including *C/ebpα* and *β*, *Pparγ*, and *Srebp1c* (38). This prior work also demonstrated *Srebp1c* mRNA concentrations correlated negatively with inflammatory cytokine levels (38).

Several genes downregulated by knockdown of *Ltbp2* are also linked to fatty acid oxidation and lipid metabolism pathways, such as *Fasn*, *Fabp4*, and adiponectin. Impaired fatty oxidation is a characteristic of HIV lipodystrophy (39, 40). Adiponectin, *Glut4*, and *Ppar*γ, integral genes to insulin action and sensitivity, were also dampened by *Ltbp2* downregulation. Insulin resistance is a common clinical finding in HIV lipodystrophy often presenting in association with other metabolic sequelae, such as ectopic fat accumulation and inflammation (10, 12, 41). While existing miRNA databases do not report *Ltbp2* as a target of our identified miRNAs, our data reveal a clear link between these miRNAs and *Ltbp2*. Knockdown of *Wisp2* also resulted in downregulation of markers of adipocyte differentiation though to a somewhat lesser degree than *Ltbp2*. In that regard, combined loss of *Ltbp2*, *Nebl*, and *Wisp2* did not have an additive effect across critical metabolic genes above that of *Ltbp2*, suggesting that these genes act in the same pathway.

Mutations in *Lmna* and *Pparγ* are well recognized in familial partial lipodystrophies and therefore could play a role in this acquired form of lipodystrophy. In this study, we determined that knockdown of *Ltbp2* and *Wisp2* resulted in significant downregulation of *Lamin C*. The *Lmna* gene encodes both lamins A and C — isoforms generated by alternative splicing of *Lmna*. *Lmna* gene mutations generally disrupt both lamin A and lamin C in partial lipodystrophy (42). Missense mutations on the *Lmna* gene, predominantly identified on exon 8, have been reported to cause a lipodystrophic phenotype (43, 44). There are reports of lamin A–specific mutations in partial lipodystrophy (43) and lamin C–specific mutations in generalized lipodystrophy (42). Interestingly, protease inhibitors have been linked to altered lamin C (45). We demonstrate reduced *Lamin C* in response to knockdown of key genes dysregulated by miRNAs in HIV lipodystrophy in cultured human preadipocytes. This occurs in vitro, independent of any ART exposure, but in vivo, it is possible that ART use may further compound this effect on *Lamin C*. The altered expression of these genes regulated by *Ltbp2* expression may contribute to fat dysregulation and subsequent metabolic complications in HIV lipodystrophy.

Finally, 3 inflammatory markers, *IL1β*, *IL6*, and *CcL20*, were increased with knockdown of *Ltbp2*. HIV lipodystrophy is associated with an inflammatory phenotype; i.e., subcutaneous adipose tissue shows an increased infiltration with inflammatory cells. Importantly, IL-6, and to a lesser extent IL-1β, have been linked to HIV lipodystrophy (46–48). Of note, knockdown of *Ltbp2* was also associated with reduced *Hmox1*. Knockdown of *Hmox1* could have an inflammatory effect as HMOX1 catalyzation can result in antiinflammatory responses by upregulation of interleukin 10 and interleukin 1 receptor antagonist expression (49, 50). Our data suggest altered miRNAs regulate *Ltbp2*, which in turn may increase inflammatory gene expression in adipose tissue, thus providing an additional mechanism for this effect. Other inflammatory markers, including *IFNβ*, were generally increased but did not reach significance with knockdown of *Ltbp2*. *Dicer* has recently been identified to repress interferons and the antiviral response in other cell types (51); thus, our data showing a trend toward increased interferon with reduced *Dicer* show a potential consistent effect in adipocytes.

This study has a number of strengths and some limitations. A strength of our study is the translational exploration of a clinical phenotype. Leveraging our preclinical model of the a*Dicer*-KO mouse, we evaluated the implications of reduced adipose Dicer expression in HIV lipodystrophy on sEV-carried miRNAs and further tested manipulation of these miRNAs, leveraging RNA-Seq to arrive at key genes contributing to critical adipogenic and inflammatory pathways. We were unable to collect additional subcutaneous adipose tissue samples in our human subjects to complement our RNA-Seq studies conducted using cultured human preadipocytes, but we validated the potential role of *Ltbp2* in a relevant HIV database of adipose gene expression (20). Our data profiling circulating sEV patterns do not allow us to discern whether reduced *Dicer* in subcutaneous adipose is directly affecting local miRNAs, and whether such altered miRNAs affect local adipogenic pathways in a paracrine fashion, distant adipose tissues, or other organs. However, our adipocyte transfection study using *Dicer* siRNA suggests the adipocyte may be directly involved in a paracrine-like effect. Future studies should now investigate this discovered miRNA signature and related target genes to determine effects on key adipogenic pathways in critical adipose depots, including browning of white adipose tissue and oxidative stress, as well as metabolic pathways in other critical organs, such as the liver, to determine how this mechanism contributes to overall metabolic dysregulation in HIV. Future studies should also assess if these pathways are important in other conditions of reduced *Dicer*, such as aging and overfeeding (9, 52), which are characterized by metabolic dysregulation.

In the current investigation, we were unable to disentangle any specific contribution of ART in reducing *Dicer* and dysregulating circulating miRNAs. Nonetheless, our experiments in adipocyte cultures were performed in the absence of any ART, suggesting that the downstream consequences of reduced *Dicer* and altered miRNAs on adipose function occur independently of ART. Further studies should investigate this question.

In summary, we conclude miR-20a-3p functions as a key marker among individuals with HIV lipodystrophy with altered adipose tissue *Dicer* expression. Overexpression of miR-20a-3p was shown to promote metabolic perturbations, potentially through the downregulation of *Ltbp2* in the adipocyte, leading to dysregulation in critical markers of adipose differentiation and inflammation, and a relevant lipodystrophy gene, *Lamin C*. As such, these studies provide a plausible mechanistic schema, whereby miR-20a-3p downregulates *Ltbp2*, leading to adipocyte dysfunction in HIV lipodystrophy. This study demonstrates a potentially novel pathway that can be targeted to improve adipose dysfunction in this population.

## Methods

### Study participants.

Eighteen participants with HIV, 9 with and 9 without lipodystrophy, and 9 uninfected participants without HIV were previously recruited in an observational study to assess *Dicer* expression in the abdominal subcutaneous adipose tissue (6). All participants were male between the ages of 18 and 60 years with a BMI 18–35.0 kg/m^2^. Among participants with HIV, ART use had been stable for more than 12 months. A single investigator assessed for HIV-related lipodystrophic changes in fat. Lipodystrophy was characterized by the presence of excess dorsocervical fat accumulation. MRI also confirmed the examination (see MRI methods below). Major exclusion criteria included hemoglobin less than 10.0 g/dL; known history of diabetes and use of antidiabetic medications; abnormal thyroid function; use of glucocorticoids, growth hormone, growth hormone–releasing hormone, or other anabolic therapies within 3 months of enrollment; current substance abuse; or active or serious chronic medical conditions other than HIV.

### Assessment of fat redistribution.

MRI of the neck was performed on a Siemens 3T Trio magnetic resonance system using phased-array neck and body matrix coils. A volumetric 3D Dixon gradient-echo multiecho pulse sequence with 6 echo times (repetition time = 20 ms; echo time = 2.46, 6.15, 9.84, 12.3, 14.76, 17.22 ms; flip angle = 5; slice thickness = 3 mm; field of view = 42 cm; matrix = 256 × 256) was employed. Sagittal images were reconstructed to identify the level of C7 vertebral body. Axial images at the level of C7 were reconstructed and used for measurement of dorsocervical adipose tissue area. Vertical reference lines were placed along the lateral border of the vertebral body of C7 and projected over dorsocervical adipose tissue, providing standardized lateral boundaries for the fat depot. Dorsocervical adipose tissue was demarcated anteriorly by the paraspinal muscles and posteriorly by the dorsocervical skin. Area measurements within these boundaries were expressed in square centimeters. In addition, MRI was acquired using an axial T1-weighted, fat-suppressed pulse sequence obtained at the level of L4 vertebral body for determination of visceral and subcutaneous fat areas utilizing commercial software (Vitrak, Merge e/Film).

### Biochemical and metabolic assessment.

Participants presented after an overnight fast for blood sampling. CD4^+^ and CD8^+^ T cell counts were assessed by flow cytometry. HIV viral load was determined by ultrasensitive real-time PCR (Roche cobas amplicor). As previously described, *Dicer* expression was determined from subcutaneous adipose tissue biopsies (6).

### Abdominal fat biopsy.

Surgical sampling of subcutaneous abdominal fat was performed under local anesthesia with 1% lidocaine, using a 4 mm diameter punch biopsy. Specimens were snap-frozen in a dry ice/ethanol bath and immediately transferred to –80°C.

### Animals.

Generation of adipocyte-specific *Dicer*-KO mice (a*Dicer*-KO; Adiponectin-Cre+ *Dicer*^fl/fl^) and control mice (Adiponectin-Cre-*Dicer*^fl/fl^) was described elsewhere (8). Female and male mice aged between 10 and 18 weeks were used for the experiments. Mice were anesthetized with Avertin (MilliporeSigma), and blood was collected by cardiac puncture.

### Human preadipocytes and white adipogenesis differentiation.

Human white preadipocytes were provided by the adipocyte core of the Boston Nutrition Obesity Research Center (BNORC). Abdominal subcutaneous fat tissue was obtained from people undergoing plastic surgery. Donors were 30–60 years old, female, of BMI range 21.7–45.7 kg/m^2^, and nondiabetic. Adipose stromal cells were isolated as previously described (14). Briefly, minced tissue was treated with collagenase solution (1 mg/mL HBSS) (type 1, Worthington Biochemical) in a continuous shaker at 37°C for 2 hours. The digested tissue was filtered through a 250 μm mesh (Component Supply, Inc.). Cells were centrifuged at 500*g* for 10 minutes at room temperature. The red blood cells in the cell pellets were lysed (0.154 mM NH_4_Cl, 10 mM K_2_HPO_4_, and 0.1 mM EDTA, pH 7.3). The washed cells were plated using α-MEM (Gibco, Thermo Fisher Scientific) with 10% FBS (Gemini Bio Products) and 100 U/mL penicillin with 10 μg/mL streptomycin (pen/strep) (Corning). For the experiments, cells were grown in DMEM high glucose with 20% FBS and 1% pen/strep until confluence. At day 0 of the differentiation protocol, cells were preinduced using growth media supplemented with 500 nM insulin; 2 nM 3,3′,5′-triiodo-l-thyronine (T3); and 1 μM rosiglitazone. This medium was renewed every 2–3 days. At day 6, cell differentiation was induced using growth medium supplemented with 500 nM insulin, 2 nM T3, 1 μM rosiglitazone, 0.54 mM 3-isobutyl-1-methylxanthine (IBMX), 33 μM biotin, 17 μM pantothenate, 0.1 μM dexamethasone, and 30 μM indomethacin. This medium was renewed every 2–3 days for 12 additional days, after which cells can be considered fully differentiated (day 18 of differentiation protocol). All chemicals and reagents were purchased from MilliporeSigma unless otherwise noted. The mimic and anti-miR transfection, with 83 nM mimic miR-20a-3p (Horizon Discovery, Dharmacon) and anti–miR-186 and anti–miR-324-5p (miRVana, Thermo Fisher Scientific) mixed with lipofectamine RNAiMAX (Thermo Fisher Scientific), was done at day 5 after starting the preinduction phase. As controls, a positive control miRIDIAN microRNA Mimic targeting aldolase-A and a miRIDIAN Dy-547–labeled miRNA Transfection Control (both from Horizon Discovery, Dharmacon) were transfected in parallel. For the transfection with siRNAs against *Ltbp2*, *Nebl*, *Wisp2*, or nontargeting control siRNA, we used 16.67 nM each single siRNA in combination with lipofectamine RNAiMAX. For the triple siRNA combination, one-third of each siRNA of the above siRNAs was used (5.55 nM each). Transfection with Dicer siRNA (Horizon Discovery, Dharmacon) was also performed at day 5 after starting the preinduction phase. One day later differentiation was induced as described above, and samples were collected for RNA isolation 6 days after differentiation induction.

### Human preadipocytes and beige adipogenesis differentiation.

Human white preadipocytes were obtained and grown until confluence as described in the previous section. After reaching confluence, cells were transfected with siRNAs against *Ltbp2*, *Nebl*, or *Wisp2* or the triple combination as done for white adipogenesis above. One day after transfection with siRNAs, beige adipogenesis was induced based on the protocol described by Singh et al. (53), with slight modifications. Briefly, confluent cells were induced to beige differentiation by adding the following reagents resuspended in DMEM/F12 (Thermo Fisher Scientific, 11965118) with 10% FBS and 1% pen/strep: 200 ng/mL recombinant hIGF-I (Peprotech, 100-11), 8 ng/mL human basic FGF (Miltenyi Biotech, 130-093-838), 100 ng/mL human BMP7 (R&D Systems, 354-BP), 10 μM Y27632 (Cayman Chemical, 10005583), 2 μM rosiglitazone (MilliporeSigma, R2408), 1 nM T3 (MilliporeSigma, T6397), 1 μM dexamethasone (MilliporeSigma, D4902), and 500 μM IBMX (MilliporeSigma, I5879). Medium was replaced every 3 days. Lipid droplet accumulation was readily observable a few days after induction and increased with time until reaching approximately 80%–90% of the plate at day 21 after induction. On this day, cells were washed with PBS and added to TRIzol for RNA isolation as described below.

### sEV isolation.

sEV isolation was performed using standard differential ultracentrifugation protocol (15) on 2–3 mL of serum from each of the 27 participants (9 HIV/lipo, 9 HIV/nonlipo, 9 non-HIV). Serum was collected in a tube without anticoagulant and underwent centrifugation at 4°C at setting 1500*g* for 10 minutes. Briefly, serum was diluted with PBS and successively centrifuged at 2000*g* for 10 minutes and 10,000*g* for 30 minutes. The supernatant was then ultracentrifuged at 100,000*g* for 70 minutes using an SW-28 rotor (Beckman-Coulter) to pellet the sEV. Pellets were washed with PBS, centrifuged again at 100,000*g* for 70 minutes, and either resuspended in PBS for nanoparticle tracking analysis, immunoblotting, nanoparticle tracking analysis, and electron microscopy or resuspended in TRIzol reagent (Thermo Fisher Scientific) for RNA isolation and subsequent miRNA profiling. For the sEV isolation of mouse serum, blood obtained by cardiac puncture sat for 30 minutes at room temperature to allow coagulation prior to centrifuging twice at 500*g* for 5 minutes and 2000*g* for 10 minutes to remove cells and cellular debris. Supernatant was mixed 1:4 with double-filtered PBS and centrifuged at 10,000*g* for 30 minutes to remove large vesicles. Supernatants containing serum sEVs were mixed in pools, each consisting of 10,000 g derived supernatants from 2 females and 1 male of the same genotype, having the same age distribution in all control and a*Dicer*-KO samples. These pools were ultracentrifuged at 100,000*g* for 70 minutes using an SW-28 rotor. The pellets containing the sEVs were washed with double-filtered PBS and centrifuged again at 100,000*g* for 70 minutes. After removing all supernatant, pellets were resuspended in TRIzol and proceeded to RNA isolation, miRNA retrotranscription, and PCR assay as described below.

### Nanoparticle tracking analysis.

Vesicle concentration and size distribution was determined by the dynamic light scattering technology using a Nanosight LM10 (Malvern Panalytical) at the Nanosight Nanoparticle Sizing & Quantification Facility at Massachusetts General Hospital (Charlestown, Massachusetts, USA). The script was programmed to take 5 videos for 30 seconds each for each sample. Samples were diluted 1:50 prior to running Nanosight system. For the calculation of the sEV concentration, this dilution and the volume of original serum where they were isolated from were taken into account.

### Immunoblotting.

Serum sEV and human 293T cells (Takara catalog 632180) were resuspended in RIPA lysis buffer (MilliporeSigma) containing 0.1% SDS and protease and phosphatase inhibitors (Biotool) and incubated on ice for 30 minutes prior to centrifugation at 12,000*g* for 10 minutes. Supernatants were used for Western blotting in SDS-PAGE electrophoresis. Protein concentration was determined by a BCA kit (Thermo Fisher Scientific). The following antibodies were used: TSG101 (sc-7964, Santa Cruz Biotechnology), CD63 (ab68418, Abcam), and calnexin (ab22595, Abcam).

### Oil red O staining.

Human adipocytes were grown, differentiated, and transfected against *Ltbp2*, *Nebl*, *Wisp2*, and the triple combination as described above. Cells were washed with PBS and fixed with 4% paraformaldehyde for 5 minutes. Cells were washed with PBS again and incubated for 1 hour with oil red O (ORO) working solution. To make ORO working solution, ORO stock solution (0.5% ORO in isopropanol) was mixed 3:2 with water and filtered. After 1-hour incubation, cells were washed with water, and representative photographs were taken immediately. Quantification of ORO signal was performed by ImageJ (NIH). Briefly, red/green/blue channels were separated in 3 pictures. Signal from red-containing pictures was quantified by selecting a 0–50 threshold, and the percentage of positive area was later calculated.

### Electron microscopy.

CD63 immunogold staining of sEV preparations was performed at Electron Microscopy Facility at Harvard Medical School. sEVs were isolated by ultracentrifugation as described above and adsorbed to a hydrophilic carbon coated grid. After blocking with BSA, grids were incubated with antibody against the sEV marker CD63 (BD Pharmingen 556019) and later an IgG secondary antibody (Abcam ab6709). The antibody complex was detected using Protein A-gold (10 nm). The grids were examined in a JEOL 1200EX transmission electron microscope, and images were recorded with an AMT 2k charge-coupled device camera.

### RNA isolation and miRNA profiling.

Upon addition of chloroform, samples were centrifuged at 12,000*g* for 15 minutes. The upper phase was collected and mixed with isopropanol, ammonium acetate (250 mM), and RNA-grade glycogen (1 μg/mL, Thermo Fisher Scientific) and incubated overnight at –20°C. Samples were then centrifuged at 12,000*g* for 30 minutes, washed twice with 75% ethanol, and resuspended in nuclease-free water. The RNA concentration was assessed by NanoDrop, and equal amounts of RNA for each sample were used for miRNA profile analysis. This was accomplished using a quantitative real-time PCR–based kit (RA660A-1, System Biosciences) following manufacturer’s instructions. For single detection of miRNAs, RNA was retrotranscribed using miRCURY LNA Starter kit (QIAGEN) and assessed by PCR using highly specific miRCURY LNA primers (QIAGEN).

RNA extraction from human adipocytes was performed as described for sEV RNA isolation. Reverse transcription was done using High Capacity Reverse transcription kit (Applied Biosystems, Thermo Fisher Scientific) following manufacturer’s instructions.

### RNA-Seq.

The total-RNA samples from human adipocytes were quantified using an Agilent 4200 TapeStation instrument, with a corresponding Agilent TapeStation RNA assay. The resulting RNA integrity number scores and concentrations were taken into account for qualifying samples to proceed.

The samples were normalized to 200 ng of input in 50 μL (4 ng/μL), and the mRNA was captured using oligo-dT beads as part of the KAPA mRNA HyperPrep workflow. cDNA synthesis, adapter ligation, and amplification were conducted subsequently as part of the same workflow. Following amplification, residual primers were eluted away using KAPA Pure Beads in a 0.63× SPRI-based cleanup.

The resulting purified libraries were run on an Agilent 4200 TapeStation instrument, with a corresponding Agilent High Sensitivity D1000 ScreenTape assay to visualize the libraries and check that the size and concentrations of the libraries matched the expected product. qPCR with the KAPA Library Quantification kit, which uses primers complementary to the sequencing flow-cell oligos, was run to confirm the functional concentration. Molarity values obtained from this assay were used to normalize all samples in equimolar ratio for 1 final pool. The pool was denatured and loaded onto an Illumina NextSeq 500 instrument, with a high-output 75-cycle kit to obtain single-end 75 bp reads. The pool was loaded at 1.6 pM, with 5% PhiX spiked in as a sequencing control. The base call files were demultiplexed through the Harvard Biopolymers Facility Genomics Core’s pipeline, and the resulting FASTQ files were used in subsequent bioinformatic analysis. RNA-Seq data were deposited in the National Center for Biotechnology Information’s Gene Expression Omnibus database under accession number GSE183822.

### Statistics.

Normality of distribution was determined using the Shapiro-Wilk test. Data are presented as mean ± SEM or median and IQR, depending on the normality of the distribution. Categorical variables are reported as proportions. Primary demographic comparisons were made between all 3 groups, non-HIV, HIV nonlipodystrophic, and HIV lipodystrophic, by overall comparison among the group by the appropriate test: 1-way ANOVA or Kruskal-Wallis test. Univariate regression was performed using Spearman’s correlation coefficient.

For miRNA profiling analysis, the data from 2 participants (1 HIV/lipo and another HIV/nonlipo) were removed due to high degree of hemolysis that significantly affected the miRNA profile results (Supplemental Table 1). As an initial step, although 1113 different miRNAs were measured, only those with Ct more than 35 in at least 5 samples per group (997 miRNAs) were considered expressed and therefore kept for further analysis. Expression data for miRNA expression were normalized to the mean Ct of all miRNAs for each sample. All samples displayed a similar Ct distribution. miRNA nomenclature was based on miR-Base database version 22 (https://www.mirbase.org). To discover differential miRNA expression among groups, we used Limma, an R package for linear modeling that powers differential expression analyses (54). The same statistical method was used for analysis of RNA-Seq data. For RNA-Seq, genes meeting criteria for an absolute fold change 1.5 and *P* < 0.05 were initially considered significant (Supplemental Tables 2–7). For multiple comparisons related to miRNA and RNA-Seq, an FDR was then applied as indicated in each experiment. Statistical significance was defined as FDR < 0.05 for sEV miRNA profiling. Data for *Ltbp2* expression in HIV nonlipodystrophic and lipodystrophic abdominal subcutaneous and dorsocervical adipose tissues were obtained from data set repository GSE28073 (20). For gene expression analysis, statistical significance was performed by 1-way ANOVA test followed by Dunnett’s test comparing each group with the control group, controlling for multiple comparisons. Gene target prediction of miRNAs was performed using TargetScan and Diana-Tools software (55, 56), selecting a score more than 85. For hierarchical cluster analysis, moderated *F* test was used to detect genes that were differentially expressed between any 2 groups (namely control, miR-20a-3p, anti–miR-186, and anti–miR-324-5p). A total of 1031 genes were selected that had FDR < 0.05 in the *F* tests. Hierarchical cluster analysis was conducted based on the Euclidean distance of these selected genes. We defined 8 clusters according to the hierarchical tree. All statistical analyses were performed using SAS JMP (version 15), SPSS (version 20, IBM), and R.

### Study approval.

All animal studies were conducted in compliance with the regulations and ethics guidelines of the NIH and were approved by the IACUC of the Joslin Diabetes Center. The human study was approved by the Mass General Brigham Institutional Review Board. All individuals provided written informed consent prior to inclusion in the study.

## Author contributions

SS designed the study, recruited patients, analyzed data, and drafted and critically reviewed the manuscript. SS was assigned as primary co–first author due to patient recruitment and conceptualization of the project. RGM designed the study, analyzed data, and drafted and critically reviewed the manuscript. MT analyzed data and critically reviewed the manuscript. KVF recruited patients and critically reviewed the manuscript. ARC analyzed data and critically reviewed the manuscript. CRK analyzed data and critically reviewed the manuscript. SKG designed the study, analyzed data, and drafted and critically reviewed the manuscript.

## Supplementary Material

Supplemental data

Supplemental tables 1-7

## Figures and Tables

**Table 1:**
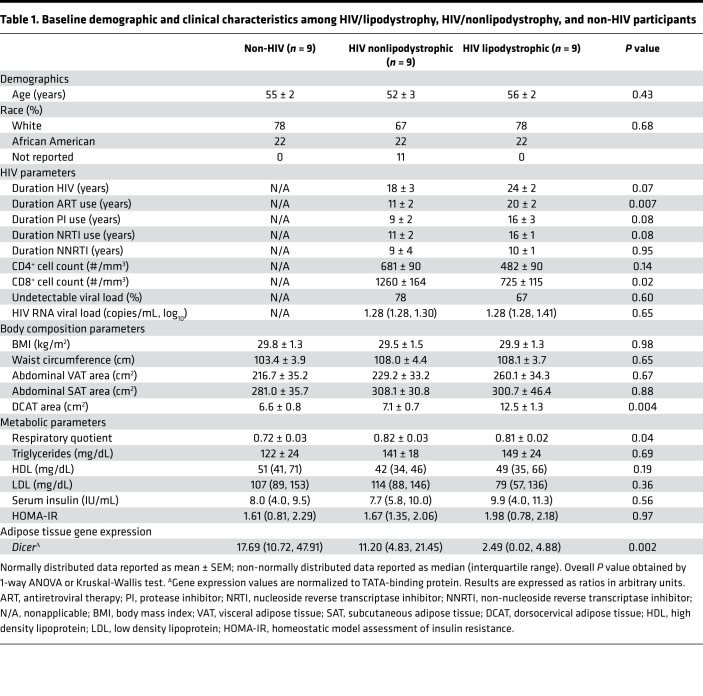
Baseline demographic and clinical characteristics among HIV/lipodystrophy, HIV/nonlipodystrophy, and non-HIV participants

**Table 2:**
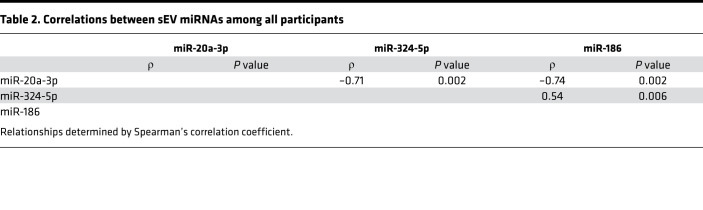
Correlations between sEV miRNAs among all participants

**Table 3:**
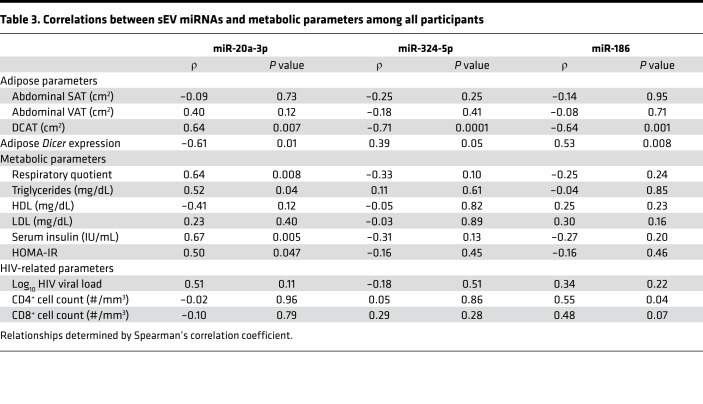
Correlations between sEV miRNAs and metabolic parameters among all participants

## Version 1 08/12/2021

In-Press Preview

## Version 2 09/22/2021

Electronic publication

## References

[1] Hussain I, Garg A (2016). Lipodystrophy syndromes. Endocrinol Metab Clin North Am.

[2] Palella FJ (2016). , et al. Anatomic fat depots and coronary plaque among human immunodeficiency virus-infected and uninfected men in the multicenter AIDS cohort study. Open Forum Infect Dis.

[3] Srinivasa S (2019). Relationship of visceral and subcutaneous adipose depots to markers of arterial injury and inflammation among individuals with HIV. AIDS.

[4] Stanley TL (2014). Effect of tesamorelin on visceral fat and liver fat in HIV-infected patients with abdominal fat accumulation: a randomized clinical trial. JAMA.

[5] Agarwal N (2013). HIV-1 Vpr induces adipose dysfunction in vivo through reciprocal effects on PPAR/GR co-regulation. Sci Transl Med.

[6] Torriani M (2016). Dysfunctional subcutaneous fat with reduced dicer and brown adipose tissue gene expression in HIV-infected patients. J Clin Endocrinol Metab.

[7] Balasubramaniam M (2018). Are microRNAs important players in HIV-1 infection? An update. Viruses.

[8] Mori MA (2014). Altered miRNA processing disrupts brown/white adipocyte determination and associates with lipodystrophy. J Clin Invest.

[9] Reis FC (2016). Fat-specific Dicer deficiency accelerates aging and mitigates several effects of dietary restriction in mice. Aging (Albany NY).

[10] Joy T (2008). Relation of body composition to body mass index in HIV-infected patients with metabolic abnormalities. J Acquir Immune Defic Syndr.

[11] Bogorodskaya M (2020). Measures of adipose tissue redistribution and atherosclerotic coronary plaque in HIV. Obesity (Silver Spring).

[12] Bourgi K (2018). Inflammation and metabolic complications in HIV. Curr HIV/AIDS Rep.

[13] Thomou T (2017). Adipose-derived circulating miRNAs regulate gene expression in other tissues. Nature.

[14] Mori MA (2019). Extracellular miRNAs: from biomarkers to mediators of physiology and disease. Cell Metab.

[15] Thery C (2006). Isolation and characterization of exosomes from cell culture supernatants and biological fluids. Curr Protoc Cell Biol.

[16] GeneCards. LTBP2 Gene (Protein Coding). Latent Transforming Growth Factor Beta Binding Protein 2. https://www.genecards.org/cgi-bin/carddisp.pl?gene=LTBP2 Accessed August 20, 2021

[17] Grunberg JR (2017). Overexpressing the novel autocrine/endocrine adipokine WISP2 induces hyperplasia of the heart, white and brown adipose tissues and prevents insulin resistance. Sci Rep.

[18] Hammarstedt A (2013). WISP2 regulates preadipocyte commitment and PPARγ activation by BMP4. Proc Natl Acad Sci U S A.

[19] Gustafson B (2013). Restricted adipogenesis in hypertrophic obesity: the role of WISP2, WNT, and BMP4. Diabetes.

[20] Sevastianova K (2011). Comparison of dorsocervical with abdominal subcutaneous adipose tissue in patients with and without antiretroviral therapy-associated lipodystrophy. Diabetes.

[21] Shackleton S (2000). LMNA, encoding lamin A/C, is mutated in partial lipodystrophy. Nat Genet.

[22] Caux F (2003). A new clinical condition linked to a novel mutation in lamins A and C with generalized lipoatrophy, insulin-resistant diabetes, disseminated leukomelanodermic papules, liver steatosis, and cardiomyopathy. J Clin Endocrinol Metab.

[23] Bidault G (2011). LMNA-linked lipodystrophies: from altered fat distribution to cellular alterations. Biochem Soc Trans.

[24] Casey Klockow L (2013). The HIV-1 protein Vpr targets the endoribonuclease Dicer for proteasomal degradation to boost macrophage infection. Virology.

[25] Bennasser Y, Jeang KT (2006). HIV-1 Tat interaction with Dicer: requirement for RNA. Retrovirology.

[26] Torriani M (2012). Deiodinase 2 expression is increased in dorsocervical fat of patients with HIV-associated lipohypertrophy syndrome. J Clin Endocrinol Metab.

[27] Cui J (2021). MicroRNA-20a-3p regulates the host immune response to facilitate the mycobacterium tuberculosis infection by targeting IKKβ/NF-κB pathway. Int Immunopharmacol.

[28] Sun JL (2020). MicroRNA regulation in hypoxic environments: differential expression of microRNAs in the liver of largemouth bass (Micropterus salmoides). Fish Physiol Biochem.

[29] Stepien EL (2018). Circulating ectosomes: determination of angiogenic microRNAs in type 2 diabetes. Theranostics.

[30] Bukhari MMM (2020). Role of MicroRNAs in establishing latency of human immunodeficiency virus. Crit Rev Eukaryot Gene Expr.

[31] Wu G (2020). MiRNA-324-5p inhibits inflammatory response of diabetic vessels by targeting CPT1A. Eur Rev Med Pharmacol Sci.

[32] Gu J (2020). Downregulated miRNA-324-5p aggravates neuronal injury induced by oxygen-glucose deprivation via modulating RAN. Exp Ther Med.

[33] Peng Y (2020). SUFU mediates EMT and Wnt/β-catenin signaling pathway activation promoted by miRNA-324-5p in human gastric cancer. Cell Cycle.

[34] Bresciani E (2019). miRNA-218 targets Lipin-1 and glucose transporter type 4 genes in 3T3-L1 cells treated with lopinavir/ritonavir. Front Pharmacol.

[35] Squillace N (2014). Changes in subcutaneous adipose tissue microRNA expression in HIV-infected patients. J Antimicrob Chemother.

[36] Valadi H (2007). Exosome-mediated transfer of mRNAs and microRNAs is a novel mechanism of genetic exchange between cells. Nat Cell Biol.

[37] Skog J (2008). Glioblastoma microvesicles transport RNA and proteins that promote tumour growth and provide diagnostic biomarkers. Nat Cell Biol.

[38] Bastard JP (2002). Association between altered expression of adipogenic factor SREBP1 in lipoatrophic adipose tissue from HIV-1-infected patients and abnormal adipocyte differentiation and insulin resistance. Lancet.

[39] Luzi L (2003). Intramyocellular lipid accumulation and reduced whole body lipid oxidation in HIV lipodystrophy. Am J Physiol Endocrinol Metab.

[40] Haugaard SB (2006). Tumor necrosis factor alpha is associated with insulin-mediated suppression of free fatty acids and net lipid oxidation in HIV-infected patients with lipodystrophy. Metabolism.

[41] van der Valk M (2001). Lipodystrophy in HIV-1-positive patients is associated with insulin resistance in multiple metabolic pathways. AIDS.

[42] Patni N (2017). Juvenile-onset generalized lipodystrophy due to a novel heterozygous missense LMNA mutation affecting lamin C. Am J Med Genet A.

[43] Speckman RA (2000). Mutational and haplotype analyses of families with familial partial lipodystrophy (Dunnigan variety) reveal recurrent missense mutations in the globular C-terminal domain of lamin A/C. Am J Hum Genet.

[44] Genschel J, Schmidt HH (2000). Mutations in the LMNA gene encoding lamin A/C. Hum Mutat.

[45] Caron M (2003). Some HIV protease inhibitors alter lamin A/C maturation and stability, SREBP-1 nuclear localization and adipocyte differentiation. AIDS.

[46] Kannisto K (2003). Expression of adipogenic transcription factors, peroxisome proliferator-activated receptor gamma co-activator 1, IL-6 and CD45 in subcutaneous adipose tissue in lipodystrophy associated with highly active antiretroviral therapy. AIDS.

[47] Lihn AS (2003). Increased expression of TNF-alpha, IL-6, and IL-8 in HALS: implications for reduced adiponectin expression and plasma levels. Am J Physiol Endocrinol Metab.

[48] Asensi V (2008). IL-1beta (+3954C/T) polymorphism could protect human immunodeficiency virus (HIV)-infected patients on highly active antiretroviral treatment (HAART) against lipodystrophic syndrome. Genet Med.

[49] Lee TS, Chau LY (2002). Heme oxygenase-1 mediates the anti-inflammatory effect of interleukin-10 in mice. Nat Med.

[50] Piantadosi CA (2011). Heme oxygenase-1 couples activation of mitochondrial biogenesis to anti-inflammatory cytokine expression. J Biol Chem.

[51] Gurung C (2021). Dicer represses the interferon response and the double-stranded RNA-activated protein kinase pathway in mouse embryonic stem cells. J Biol Chem.

[52] Mori MA (2012). Role of microRNA processing in adipose tissue in stress defense and longevity. Cell Metab.

[53] Singh AM (2020). Human beige adipocytes for drug discovery and cell therapy in metabolic diseases. Nat Commun.

[54] Ritchie ME (2006). Empirical array quality weights in the analysis of microarray data. BMC Bioinformatics.

[55] Agarwal V (2015). Predicting effective microRNA target sites in mammalian mRNAs. Elife.

[56] Paraskevopoulou MD (2013). DIANA-microT web server v5.0: service integration into miRNA functional analysis workflows. Nucleic Acids Res.

