# Supplementary material for: Altered pattern of circulating miRNAs in HIV lipodystrophy perturbs key adipose differentiation and inflammation pathways
Source: JCI Insight. 2021 Sep 22;6(18):e150399. doi: 10.1172/jci.insight.150399 (PMC8492307; doi:10.1172/jci.insight.150399)
Supplement: Supplemental data [file jciinsight-6-150399-s192.pdf]

Supplemental Figure 1

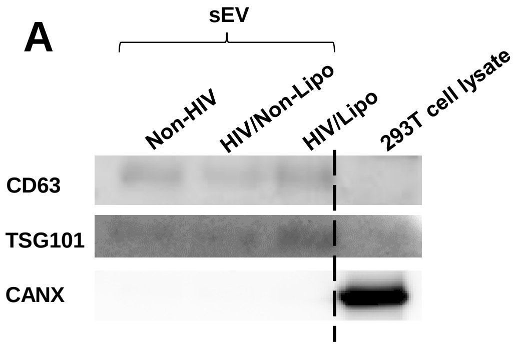

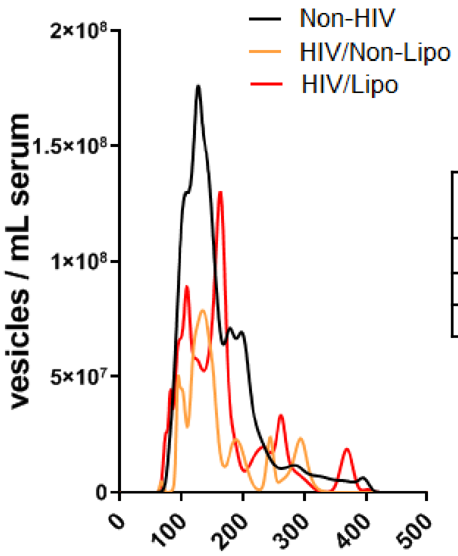

Group	sEV diameter (nm)	sEV concentration (vesicles/mL serum)
**Non-HIV**	164.1 ± 4.3	1.59 $x10^9$
**HIV/Non-Lipo**	164.8 ± 11.0	0.58 $x10^9$
**HIV/Lipo**	167.1 ± 6.4	0.99 $x10^9$

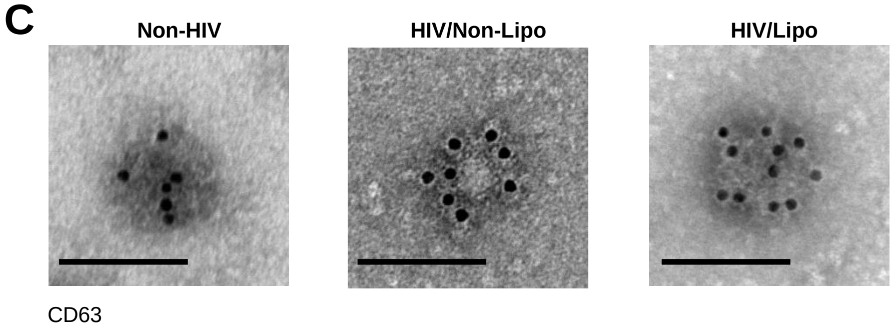

Supplemental Figure 1: Characterization of the isolated circulating sEV.

A) Exosomes were isolated from serum of HIV/Lipodystrophy, HIV/Non-Lipodystrophy, and Non- HIV participants and subjected to immunoblotting for exosomal markers CD63 and TSG101 and cellular marker CANX. A cell lysate from 293T human cell line was also included as a control.

B) Average size and concentration of the vesicles isolated from the indicated groups as well as the size distribution of the vesicles.

C) Representative example of immune electron microscopy images for CD63 from exosomes isolated from the indicated groups.

Scale bar: 100 nm.

Supplemental Figure 2

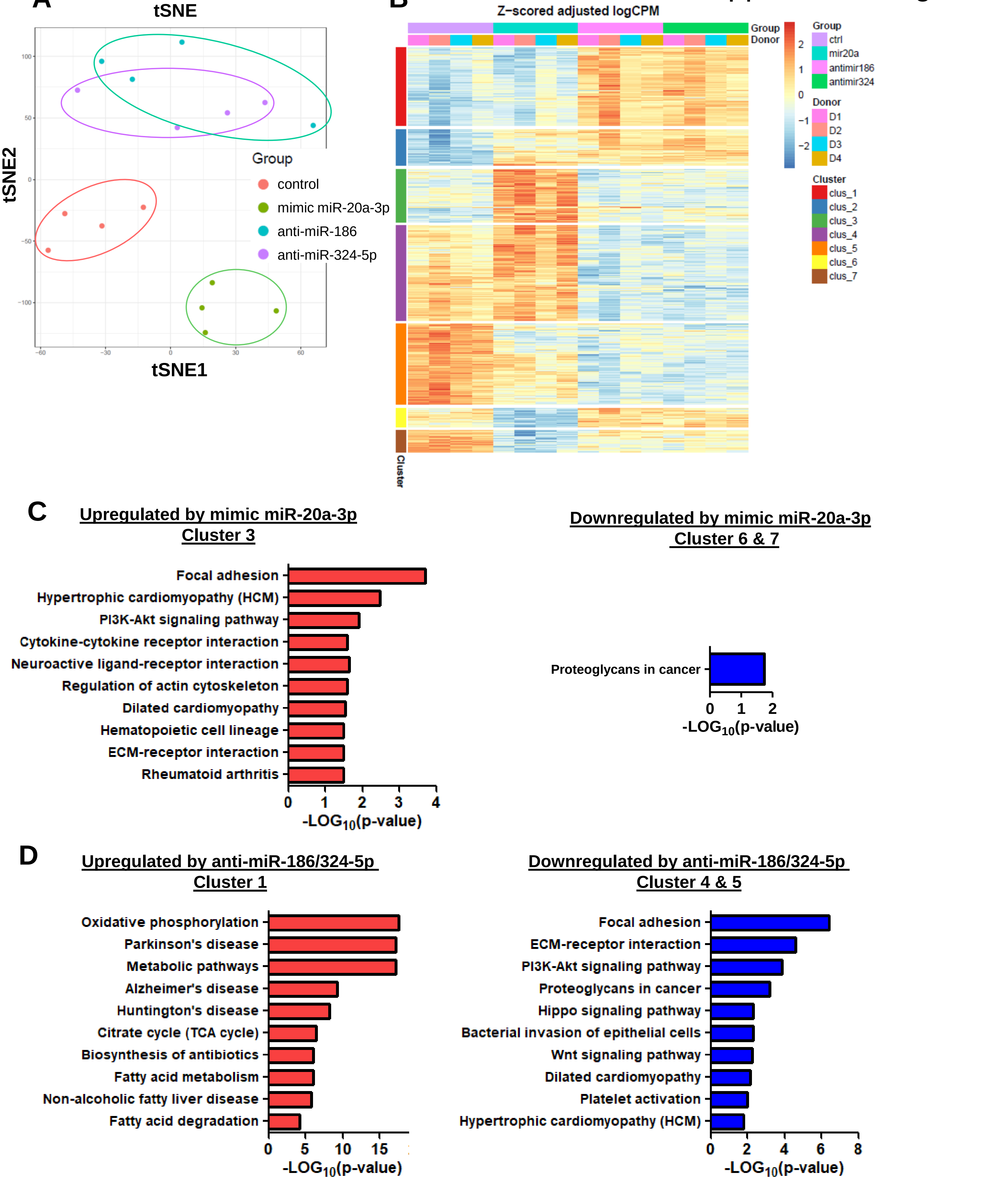

Supplemental Figure 2: Additional information on the RNAseq from Main Figure 2:

A) tSNE plot for control, mimic miR-20a-3p, anti-miR-186 and anti-miR-324.

B) Cluster analysis for the differentially expressed genes from this RNAseq for the indicated groups above. The different clusters are indicated in the vertical bar on the left.

C) Upregulated (left, correspond to cluster 3 from B) and downregulated (right, correspond to clusters 6 and 7 from B) pathways induced by treatment with mimic miR-20a-3p.

D) Upregulated (left, correspond to cluster 1 from B) and downregulated (right, correspond to cluster 4 and 5 from B) pathways induced by treatment with anti-miR-186 and anti-miR-324.

n=4 in all figure panels. FDR<0.05 for hierarchical clustering analysis.

Knockdown efficiency

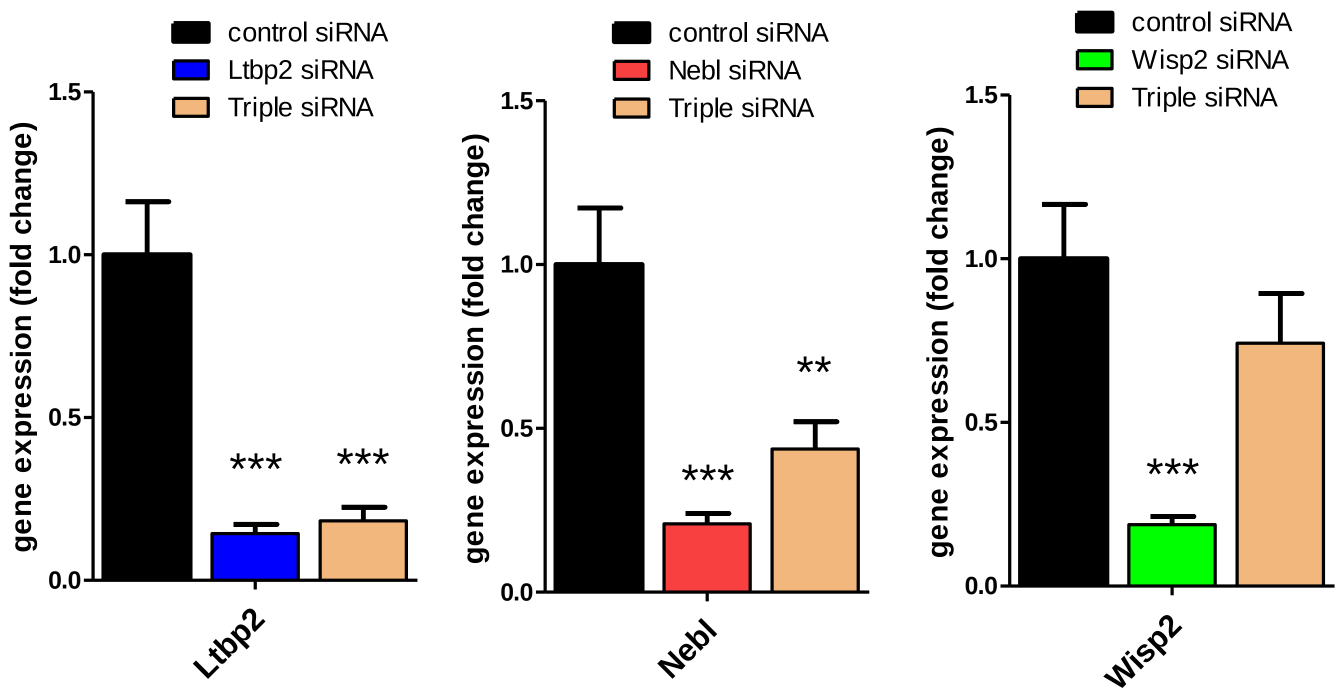

Supplemental Figure 3: Knock-down efficiency of experiment shown in Figure 3. Gene expression for *Ltbp2* (left), *Nebl* (middle) and *Wisp2* (right) by using the indicated siRNAs.

**P<0.01, ***P<0.001. n=8 in all panels, statistical comparisons was performed by Kruskal-Wallis test followed by individual Mann-Whitney U for individual comparisons

Supplemental Figure 4

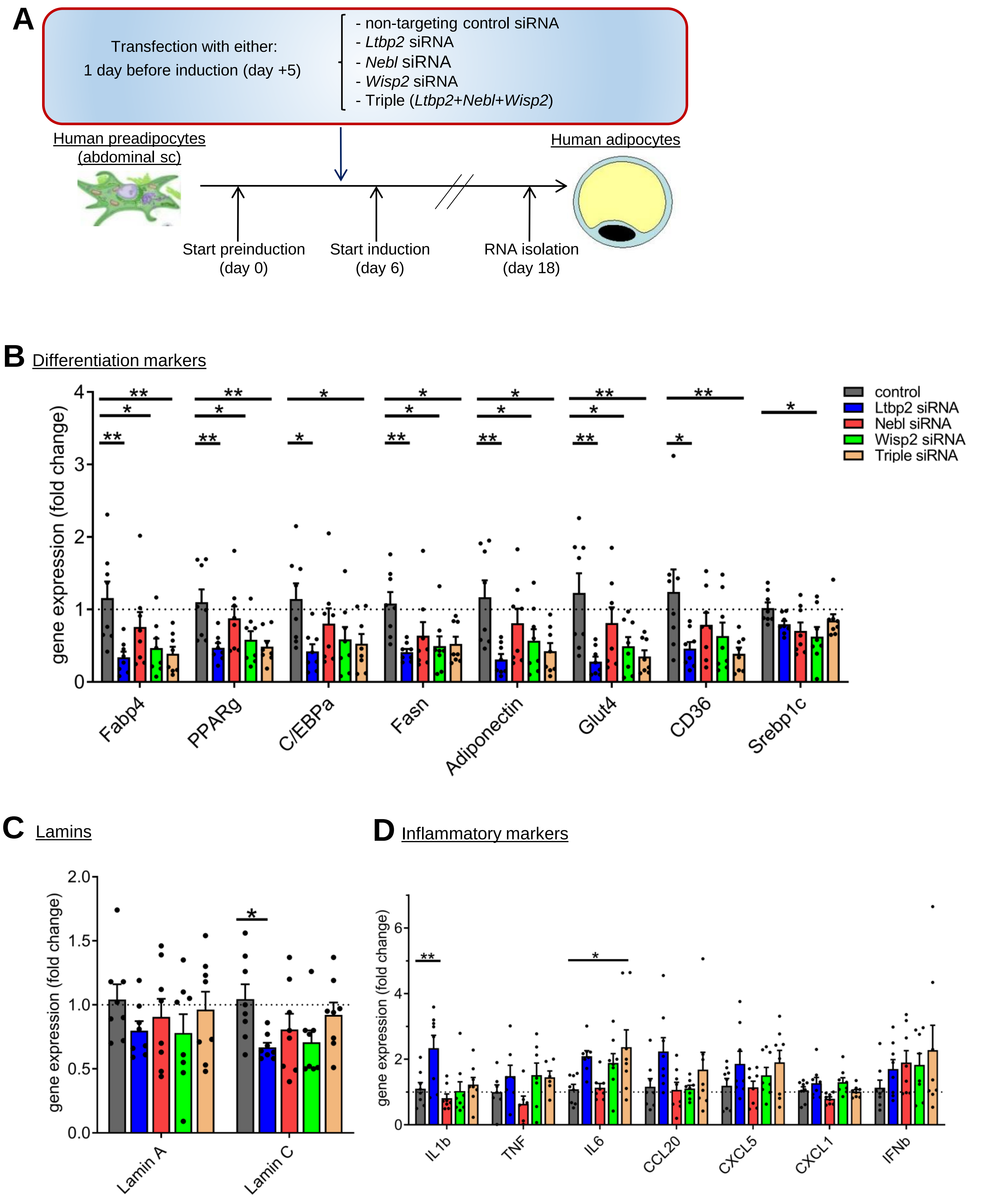

Supplemental Figure 4: *Ltbp2* regulates adipocyte differentiation, *Lamin C* expression and inflammation.

A) Diagram representing the experimental setup.

B) Effect of the treatment of preadipocytes with negative control-, *Ltbp2*-, *Nebl*-, *Wisp2*- and triple siRNA on the indicated differentiation markers.

C) Effects of the indicated treatments on the expression of *Lamin A* and *Lamin C*.

D) Effects of the indicated treatments on the expression of the inflammatory markers shown below.

Data are expressed as mean ± SEM. *P<0.05, **<0.01. n=8 in all panels, statistical comparisons in B-D was performed by ANOVA followed by Dunnett's Test comparing individual groups to control.

Supplemental Figure 5

A

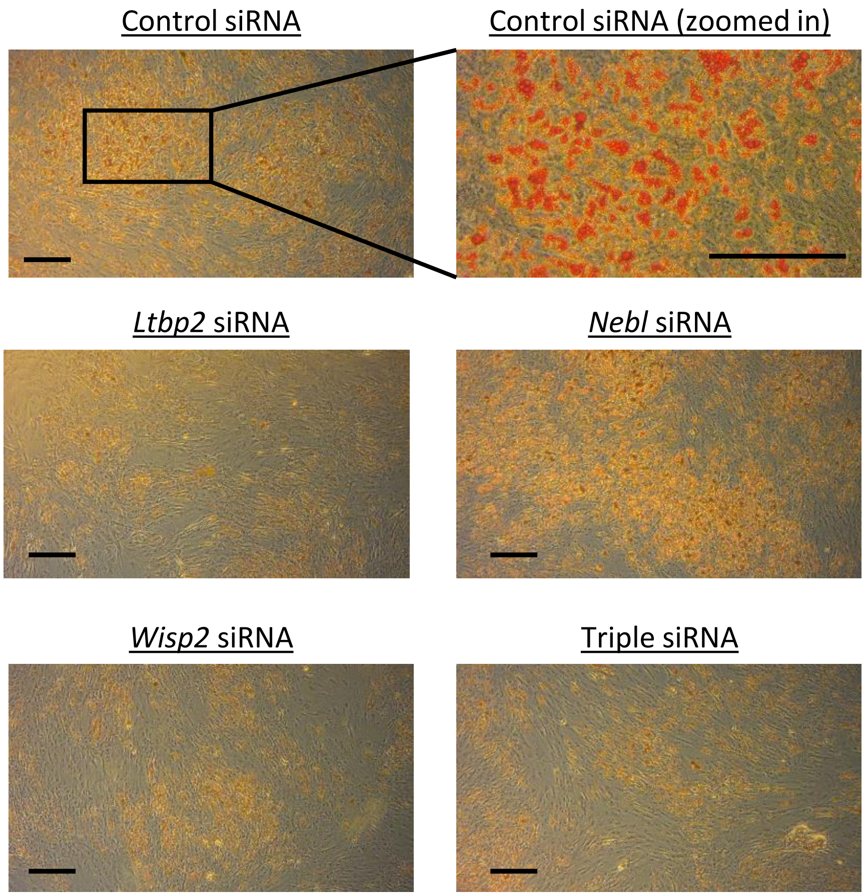

B

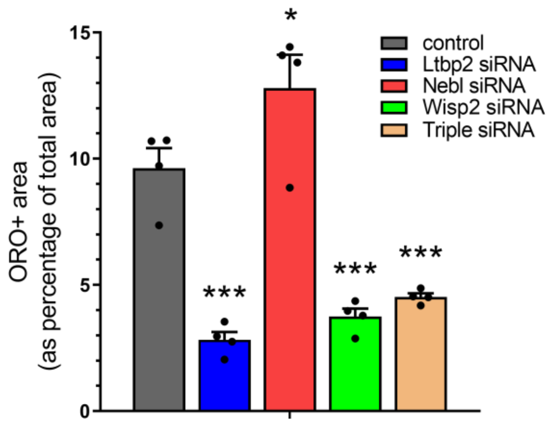

Supplemental Figure 5: Knockdown of *Ltbp2* demonstrates to reduced adipocyte differentiation

A) Histologic images of adipocytes treated with *Ltbp2*, *Nebl*, *Wisp2* and triple siRNA stained with oil-red-O.

B) Quantitative measure of oil-red-O staining as a percentage of total area
Scale bar: 100 μm.

n=4, data are expressed as mean ± SEM. *P<0.05, statistical comparisons was performed by ANOVA followed by Dunnett’s Test comparing individual groups to control.
.

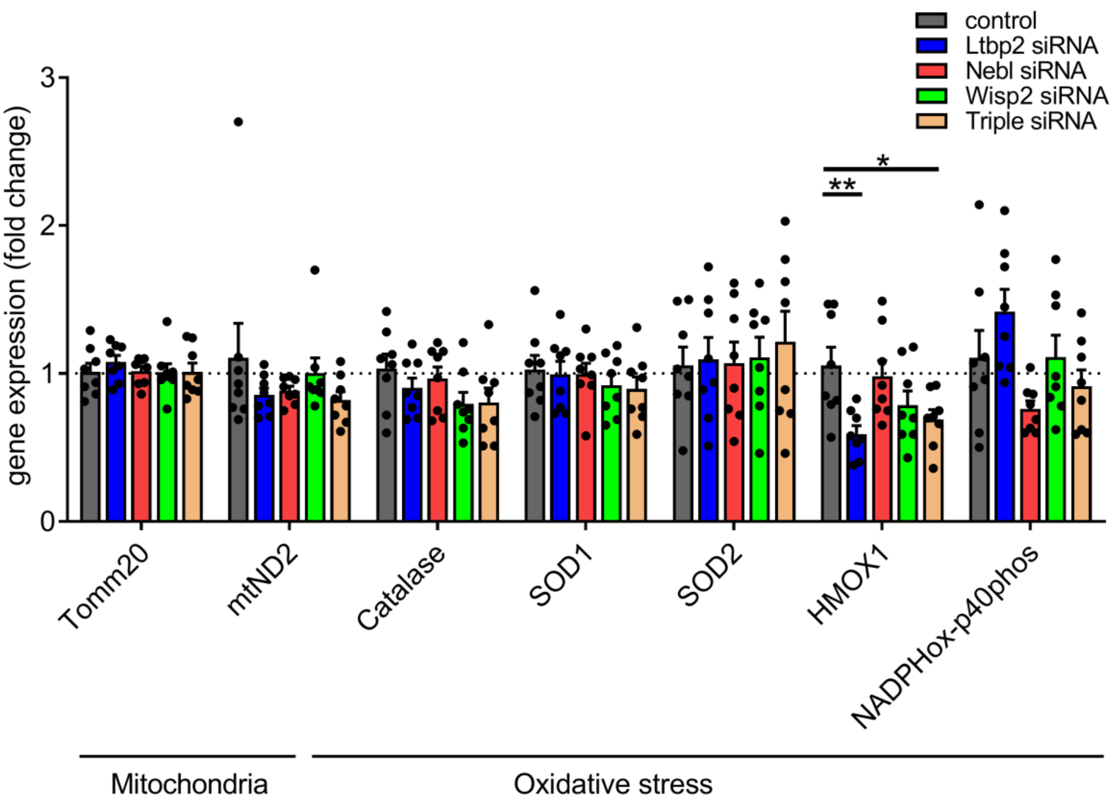

Supplemental Figure 6: *Ltbp2* regulates *Hmox1*, a marker of oxidative stress, in adipocytes

Effect of the treatment of preadipocytes with negative control-, *Ltbp2*-, *Nebl*-, *Wisp2*- and triple siRNA on the expression of mitochondrial and oxidative stress markers.

Data are expressed as mean ± SEM. *P<0.05, **<0.01. n=8 in all panels, statistical comparisons was performed by ANOVA followed by Dunnett's Test comparing individual groups to control.

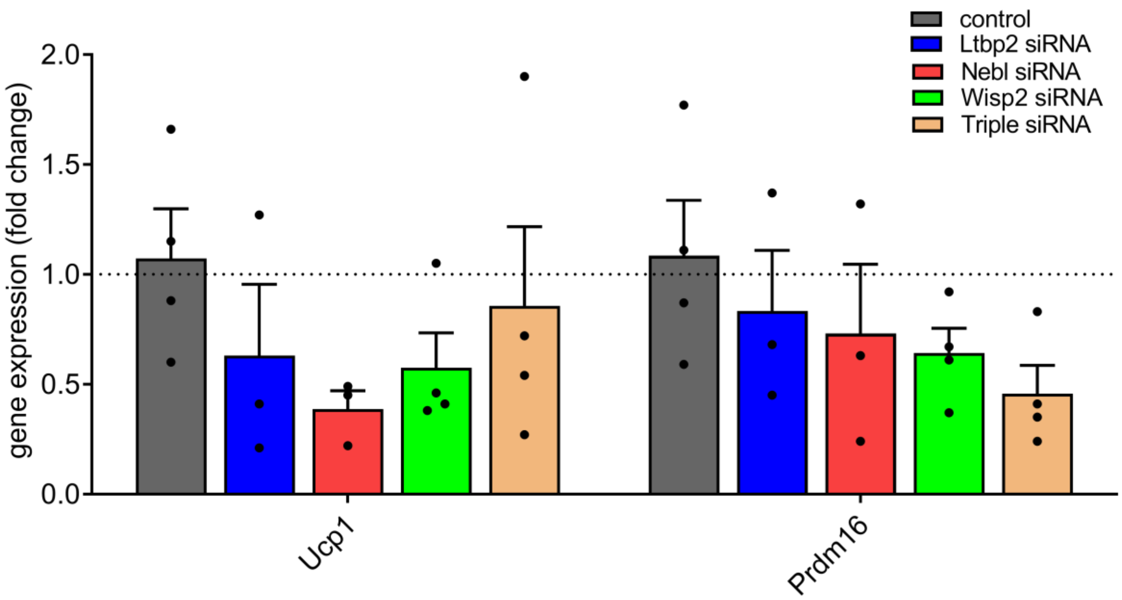

Supplemental Figure 7: Exploration of a beige adipocyte differentiation protocol
Effect of the treatment of preadipocytes with negative control-, *Ltbp2*-, *Nebl*-, *Wisp2*- and triple siRNA on the expression of brown and beige adipose tissue markers.

Data are expressed as mean ± SEM. *P<0.05. n=4 in all panels, statistical comparisons was performed by ANOVA followed by Dunnett’s Test comparing individual groups to control.
