# Supplementary material for: Altered pattern of circulating miRNAs in HIV lipodystrophy perturbs key adipose differentiation and inflammation pathways
Source: JCI Insight. 2021 Sep 22;6(18):e150399. doi: 10.1172/jci.insight.150399 (PMC8492307; doi:10.1172/jci.insight.150399)
Supplement: Supplemental tables 1-7 [file jciinsight-6-150399-s193.pdf]

**Supplemental Table 1.** Small extracellular vesicles miRNA Profile Among HIV/Lipodystrophy, HIV/Non-Lipodystrophy, and Non-HIV participants				
**Subject Classification**	**miRNA Measured**	**miRNA Detected**	**miRNA ct<35**	**Average ct**
**Non-HIV**	1113	913	643	33.48566265
**Non-HIV**	1113	953	715	33.18066107
**Non-HIV**	1113	949	703	33.31944152
**Non-HIV**	1113	799	531	33.67141427
**Non-HIV**	1113	977	792	32.42082907
**Non-HIV**	1113	1038	868	32.26423892
**Non-HIV**	1113	936	669	33.43976496
**Non-HIV**	1113	979	737	33.1180286
**Non-HIV**	1113	1022	892	31.96048924
**HIV/non-lipodystrophy**	1113	1039	879	32.11861405
**HIV/non-lipodystrophy**	1113	1058	944	31.29917769
**HIV/non-lipodystrophy**	1113	1038	891	32.01327553
**HIV/non-lipodystrophy**	1113	835	570	33.50558084
**HIV/non-lipodystrophy**	1113	1058	969	30.97307183
**HIV/non-lipodystrophy**	1113	985	788	32.67791878
**HIV/non-lipodystrophy**	1113	1013	855	32.33650543
**HIV/non-lipodystrophy**[A]	1113	705	430	33.85049645
**HIV/non-lipodystrophy**	1113	875	583	33.47907429
**HIV/lipodystrophy**	1113	793	483	34.14481715
**HIV/lipodystrophy**	1113	979	751	33.01707865
**HIV/lipodystrophy**	1113	899	609	33.79969967
**HIV/lipodystrophy**	1113	1055	954	31.57533649
**HIV/lipodystrophy**	1113	965	700	33.21749223

**HIV/lipodystrophy**	1113	851	524	33.99361927
**HIV/lipodystrophy**	1113	979	810	32.59437181
**HIV/lipodystrophy**	1113	894	622	33.45416107
**HIV/lipodystrophy**[A]	1113	526	265	34.53249049

[A]Outliers removed from analysis

**Supplemental Table 2. Gene targets downregulated by all miRNAs in transfected human adipocytes**			
	**miRNA-20a-3p mimic**	**anti-miRNA-186**	**anti-miRNA-324-5p**
**LTBP2**	-1	-1	-1
**MFAP2**	-1	-1	-1
**COL6A3**	-1	-1	-1
**HIF3A**	-1	-1	-1
**SFRP2**	-1	-1	-1
**CMKLR1**	-1	-1	-1
**HMCN1**	-1	-1	-1
**KIAA1217**	-1	-1	-1
**ADGRB1**	-1	-1	-1
**TMEM132B**	-1	-1	-1
**BDKRB2**	-1	-1	-1
**GAS7**	-1	-1	-1
**NLGN4X**	-1	-1	-1
**ARL4C**	-1	-1	-1
**ARHGEF19**	-1	-1	-1
**FBXL7**	-1	-1	-1
**EMP3**	-1	-1	-1
**RNF144A**	-1	-1	-1
**WISP2**	-1	-1	-1
**GALNT15**	-1	-1	-1
**CDH11**	-1	-1	-1
**SHISAL1**	-1	-1	-1
**HECW2**	-1	-1	-1
**PPL**	-1	-1	-1

**NEBL**	-1	-1	-1
**CILP2**	-1	-1	-1
**CYP27A1**	-1	-1	-1
**PTGFR**	-1	-1	-1
**DNM1**	-1	-1	-1
**FMNL3**	-1	-1	-1
**RUNX2**	-1	-1	-1
**TIAM1**	-1	-1	-1
**GEM**	-1	-1	-1
**KCNK2**	-1	-1	-1
**SLC38A5**	-1	-1	-1
**ADAM22**	-1	-1	-1
**SCARA3**	-1	-1	-1
**AC009093.1**	-1	-1	-1
**KCNJ12**	-1	-1	-1
**DPT**	-1	-1	-1
**CPNE7**	-1	-1	-1
**GDPD3**	-1	-1	-1
**PDPN**	-1	-1	-1
**GFRA1**	-1	-1	-1
**PLEKHG4**	-1	-1	-1
**SCARA5**	-1	-1	-1
**PTCHD4**	-1	-1	-1
**IRX1**	-1	-1	-1
**PTGIS**	-1	-1	-1
**WNT5A**	-1	-1	-1
**HTRA3**	-1	-1	-1

**GPR35**	-1	-1	-1
**TNFRSF19**	-1	-1	-1

**Supplemental Table 3. Gene targets upregulated by all miRNAs in transfected human adipocytes**			
	**miRNA-20a-3p mimic**	**anti-miRNA-186**	**anti-miRNA-324-5p**
**RRAS2**	1	1	1
**RCAN1**	1	1	1
**MAOA**	1	1	1
**BMS1P10**	1	1	1
**CANX**	1	1	1
**RET**	1	1	1
**CRABP1**	1	1	1

**Supplemental Table 4. Gene targets downregulated by anti-miRNA-186 and miRNA-324-5p in transfected human adipocytes**			
	**miRNA-20a-3p mimic**	**anti-miRNA-186**	**anti-miRNA-324-5p**
**DEPTOR**	0	-1	-1
**SERPINE1**	0	-1	-1
**IL4R**	0	-1	-1
**CCND2**	0	-1	-1
**COL1A1**	0	-1	-1
**STC2**	0	-1	-1
**ABCC3**	0	-1	-1
**PTGS1**	0	-1	-1
**TMEM155**	0	-1	-1
**MRC2**	0	-1	-1
**HEG1**	0	-1	-1
**SETBP1**	0	-1	-1
**IGF2**	0	-1	-1
**CXCL3**	0	-1	-1
**PAPPA**	0	-1	-1
**CNR1**	0	-1	-1
**LRRN2**	0	-1	-1
**IL1RL1**	0	-1	-1
**VDR**	0	-1	-1
**PDE7B**	0	-1	-1
**TSPAN5**	0	-1	-1
**ITGA11**	0	-1	-1
**CDH13**	0	-1	-1
**CCL2**	0	-1	-1

**MTCL1**	0	-1	-1
**CREB3L1**	0	-1	-1
**ARAP3**	0	-1	-1
**KCTD12**	0	-1	-1
**PLCB4**	0	-1	-1
**COL5A1**	0	-1	-1
**FBLN1**	0	-1	-1
**PDE1A**	0	-1	-1
**UNC5B**	0	-1	-1
**FAM167A**	0	-1	-1
**KSR1**	0	-1	-1
**FIBIN**	0	-1	-1
**TIAM1**	0	-1	-1
**GAS6-DT**	0	-1	-1
**MFAP4**	0	-1	-1
**CORO2B**	0	-1	-1
**TGFBI**	0	-1	-1
**GAS6**	0	-1	-1
**GPR161**	0	-1	-1
**TMEM44**	0	-1	-1
**GPR173**	0	-1	-1
**ZNF521**	0	-1	-1
**TNFRSF1B**	0	-1	-1
**CLSTN3**	0	-1	-1
**GALNT5**	0	-1	-1
**GFPT2**	0	-1	-1
**SCARA3**	0	-1	-1

**MT1X**	0	-1	-1
**COL7A1**	0	-1	-1
**SPOCK1**	0	-1	-1
**AFF2**	0	-1	-1
**ZNF469**	0	-1	-1
**STMN2**	0	-1	-1
**SLC12A8**	0	-1	-1
**C1RL-AS1**	0	-1	-1
**IL18R1**	0	-1	-1
**SHC3**	0	-1	-1
**MLPH**	0	-1	-1
**FAM43A**	0	-1	-1
**NCAM2**	0	-1	-1
**ERFE**	0	-1	-1
**MAPK13**	0	-1	-1
**TNFRSF10C**	0	-1	-1
**COL11A1**	0	-1	-1
**TMEM204**	0	-1	-1
**GFRA1**	0	-1	-1
**CACNA1G**	0	-1	-1
**PTCHD4**	0	-1	-1
**PRICKLE1**	0	-1	-1
**WNT5A**	0	-1	-1
**EPHA3**	0	-1	-1
**SLC9A5**	0	-1	-1
**PIP5KL1**	0	-1	-1
**GLT8D2**	0	-1	-1

**SORCS2**	0	-1	-1
**CLDN11**	0	-1	-1
**PLAU**	0	-1	-1
**RORB**	0	-1	-1
**ELMO3**	0	-1	-1
**ADAM33**	0	-1	-1
**ELFN1**	0	-1	-1
**GPM6B**	0	-1	-1
**IL21R**	0	-1	-1
**PTGIR**	0	-1	-1
**TENM2**	0	-1	-1
**AP003716.1**	0	-1	-1
**ENPP5**	0	-1	-1
**C11orf87**	0	-1	-1
**AC144831.1**	0	-1	-1

**Supplemental Table 5. Gene targets upregulated by anti-miRNA-186 and miRNA-324-5p in transfected adipocytes**			
	**miRNA-20a-3p mimic**	**anti-miRNA-186**	**anti-miRNA-324-5p**
**AKAP12**	0	1	1
**SFRP4**	0	1	1
**MRPS35**	0	1	1
**NDUFB5**	0	1	1
**SEMA3G**	0	1	1
**ATP9A**	0	1	1
**ACSL5**	0	1	1
**CKMT1A**	0	1	1
**ZNF426-DT**	0	1	1
**SPATA9**	0	1	1
**LINC00847**	0	1	1
**LBP**	0	1	1
**CACUL1**	0	1	1
**CRYAB**	0	1	1
**PYGL**	0	1	1
**NGF-AS1**	0	1	1
**AOC2**	0	1	1
**ELOVL5**	0	1	1
**IGFBP5**	0	1	1
**DECR1**	0	1	1
**OSBPL11**	0	1	1
**CANX**	0	1	1
**RETSAT**	0	1	1
**REEP6**	0	1	1

**VKORC1L1**	0	1	1
**AQP7P1**	0	1	1
**RXRG**	0	1	1
**FZD4**	0	1	1
**PIK3C2B**	0	1	1

Supplemental Table 6. Gene targets downregulated by miRNA-20a-3p mimic in transfected human adipocytes			
	**miRNA-20a-3p mimic**	**anti-miRNA-186**	**anti-miRNA-324-5p**
**LIN52**	-1	0	0
**MTMR4**	-1	0	0
**FRAS1**	-1	0	0
**VIT**	-1	0	0
**DCN**	-1	0	0
**DCLK1**	-1	0	0
**LUM**	-1	0	0
**AP001267.5**	-1	0	0
**MMP2**	-1	0	0
**SOBP**	-1	0	0
**SCD5**	-1	0	0
**MFAP5**	-1	0	0
**RIPOR2**	-1	0	0
**COMP**	-1	0	0
**ABCA9**	-1	0	0
**SNED1**	-1	0	0
**DGCR6**	-1	0	0
**CNTN3**	-1	0	0
**IFI44**	-1	0	0
**LINC00310**	-1	0	0
**NGFR**	-1	0	0
**APCDD1**	-1	0	0
**AC124242.1**	-1	0	0
**GALNT13**	-1	0	0

**EYA2**	-1	0	0
**FAM78A**	-1	0	0
**DCHS2**	-1	0	0
**CHL1**	-1	0	0
**IGF1**	-1	0	0
**UNC5C**	-1	0	0
**MMP7**	-1	0	0
**HPD**	-1	0	0
**OAS2**	-1	0	0
**C6orf47**	-1	0	0
**SPON1**	-1	0	0
**GSTM5**	-1	0	0
**CYGB**	-1	0	0
**AC073621.1**	-1	0	0
**HTR7**	-1	0	0
**FNDC1**	-1	0	0
**USP18**	-1	0	0
**LRRC4C**	-1	0	0
**NINJ2**	-1	0	0
**EFS**	-1	0	0
**DAB2IP**	-1	0	0
**Z83844.3**	-1	0	0
**MEDAG**	-1	0	0
**YPEL4**	-1	0	0
**GRID1**	-1	0	0
**JAM2**	-1	0	0
**LEPR**	-1	0	0

**RFX8**	-1	0	0
**SOX9**	-1	0	0
**LINC00475**	-1	0	0
**LINC00511**	-1	0	0

**Supplemental Table 7. Gene targets upregulated by miRNA-20a-3p mimic in transfected human adipocytes**			
	**miRNA-20a-3p mimic**	**anti-miRNA-186**	**anti-miRNA-324-5p**
**TAGLN**	1	0	0
**F2R**	1	0	0
**TP53I11**	1	0	0
**PCDH7**	1	0	0
**SAA1**	1	0	0
**NID2**	1	0	0
**AL590004.3**	1	0	0
**HSPA2**	1	0	0
**SORBS2**	1	0	0
**FLNB**	1	0	0
**ALDH1A1**	1	0	0
**CCDC80**	1	0	0
**NEDD9**	1	0	0
**ACTA2**	1	0	0
**CCDC3**	1	0	0
**ITGA1**	1	0	0
**CCL20**	1	0	0
**FBLIM1**	1	0	0
**PREX2**	1	0	0
**TRPC6**	1	0	0
**PIEZO2**	1	0	0
**HOXC13**	1	0	0
**CD1D**	1	0	0
**TNS3**	1	0	0

**ADGRF5**	1	0	0
**CXCL1**	1	0	0
**IL6**	1	0	0
**SUSD2**	1	0	0
**SEL1L3**	1	0	0
**CFHR1**	1	0	0
**ADAMTS1**	1	0	0
**EDN1**	1	0	0
**MYL9**	1	0	0
**NFASC**	1	0	0
**COL4A4**	1	0	0
**ADM**	1	0	0
**HAND2**	1	0	0
**GDF7**	1	0	0
**TFPI2**	1	0	0
**KCNK6**	1	0	0
**C1orf198**	1	0	0
**RERG**	1	0	0
**CRIM1**	1	0	0
**KRT7**	1	0	0
**A2M**	1	0	0
**OLFML2A**	1	0	0
**ACTC1**	1	0	0
**SYNE2**	1	0	0
**FILIP1L**	1	0	0
**MAP2**	1	0	0
**ADRA2A**	1	0	0

**SSTR1**	1	0	0
**CYP1A1**	1	0	0
**SLIT2**	1	0	0
**MGP**	1	0	0
**TGM2**	1	0	0
**S1PR3**	1	0	0
**FLT1**	1	0	0
**CNN1**	1	0	0
**TMEM158**	1	0	0
**IL7R**	1	0	0
**INMT**	1	0	0
**CXCL5**	1	0	0
**TET3**	1	0	0
**NT5E**	1	0	0
**LIMS2**	1	0	0
**CHST14**	1	0	0
**RGS4**	1	0	0
**RARB**	1	0	0
**TNFRSF11B**	1	0	0
**INPP4B**	1	0	0
**ANO3**	1	0	0
**EGFL7**	1	0	0
**MEGF6**	1	0	0
**PRL**	1	0	0
**HSPB7**	1	0	0
**SCUBE3**	1	0	0
**NTN4**	1	0	0

**HOXC12**	1	0	0
**HOXC13-AS**	1	0	0
**JPH2**	1	0	0
**NPY1R**	1	0	0
**LRCH2**	1	0	0
**TINAGL1**	1	0	0
**TRIM16L**	1	0	0
**ANKRD1**	1	0	0
**FAM83G**	1	0	0
**AEBP1**	1	0	0
**TNIK**	1	0	0
**MGLL**	1	0	0
**KCNS2**	1	0	0
**TMEM176B**	1	0	0
**NRK**	1	0	0
**C12orf75**	1	0	0
**GNG11**	1	0	0
**GRIN2B**	1	0	0
**LAMA5**	1	0	0
**MYH11**	1	0	0
**ITGA3**	1	0	0
**RIMS3**	1	0	0
**EMILIN2**	1	0	0
**MRVI1**	1	0	0
**CLEC3B**	1	0	0
**PLD6**	1	0	0

**TMEM236**	1	0	0
**SYPL2**	1	0	0
**WFDC1**	1	0	0
**AC068547.1**	1	0	0
**UGCG**	1	0	0
**BMP4**	1	0	0
**ATF3**	1	0	0
